# Epigenetic dynamics of human spermatogenesis and their dysregulation in non-obstructive azoospermia

**DOI:** 10.1093/procel/pwag033

**Published:** 2026-05-22

**Authors:** Haoling Xie, Jingyi Li, Li Zhang, Xueqiang Xu, Zhenhuan Jiang, Youran Cao, Lu Wen, Jie Qiao, Fuchou Tang

**Affiliations:** Biomedical Pioneering Innovation Center, Center for Reproductive Medicine, Department of Obstetrics and Gynecology, Peking University Third Hospital, Beijing 100191, China; Academy for Advanced Interdisciplinary Studies, Peking University, Beijing 100871, China; Key Laboratory of Cell Proliferation and Regulation Biology, Ministry of Education, College of Life Sciences, Beijing Normal University, Beijing 100875, China; Beijing Advanced Innovation Center for Genomics (ICG), Ministry of Education Key Laboratory of Cell Proliferation and Differentiation, Beijing 100871, China; Biomedical Pioneering Innovation Center, Center for Reproductive Medicine, Department of Obstetrics and Gynecology, Peking University Third Hospital, Beijing 100191, China; Academy for Advanced Interdisciplinary Studies, Peking University, Beijing 100871, China; Beijing Advanced Innovation Center for Genomics (ICG), Ministry of Education Key Laboratory of Cell Proliferation and Differentiation, Beijing 100871, China; Biomedical Pioneering Innovation Center, Center for Reproductive Medicine, Department of Obstetrics and Gynecology, Peking University Third Hospital, Beijing 100191, China; Key Laboratory of Assisted Reproduction (Peking University), Ministry of Education, Beijing 100191, China; Biomedical Pioneering Innovation Center, Center for Reproductive Medicine, Department of Obstetrics and Gynecology, Peking University Third Hospital, Beijing 100191, China; Academy for Advanced Interdisciplinary Studies, Peking University, Beijing 100871, China; Beijing Advanced Innovation Center for Genomics (ICG), Ministry of Education Key Laboratory of Cell Proliferation and Differentiation, Beijing 100871, China; Biomedical Pioneering Innovation Center, Center for Reproductive Medicine, Department of Obstetrics and Gynecology, Peking University Third Hospital, Beijing 100191, China; Academy for Advanced Interdisciplinary Studies, Peking University, Beijing 100871, China; Beijing Advanced Innovation Center for Genomics (ICG), Ministry of Education Key Laboratory of Cell Proliferation and Differentiation, Beijing 100871, China; Peking-Tsinghua Center for Life Sciences, Academy for Advanced Interdisciplinary Studies, Peking University, Beijing 100871, China; Key Laboratory of Cell Proliferation and Regulation Biology, Ministry of Education, College of Life Sciences, Beijing Normal University, Beijing 100875, China; Biomedical Pioneering Innovation Center, Center for Reproductive Medicine, Department of Obstetrics and Gynecology, Peking University Third Hospital, Beijing 100191, China; Beijing Advanced Innovation Center for Genomics (ICG), Ministry of Education Key Laboratory of Cell Proliferation and Differentiation, Beijing 100871, China; Biomedical Pioneering Innovation Center, Center for Reproductive Medicine, Department of Obstetrics and Gynecology, Peking University Third Hospital, Beijing 100191, China; Key Laboratory of Assisted Reproduction (Peking University), Ministry of Education, Beijing 100191, China; Academy for Advanced Interdisciplinary Studies, Peking University, Beijing 100871, China; Beijing Advanced Innovation Center for Genomics (ICG), Ministry of Education Key Laboratory of Cell Proliferation and Differentiation, Beijing 100871, China; Peking-Tsinghua Center for Life Sciences, Academy for Advanced Interdisciplinary Studies, Peking University, Beijing 100871, China; Biomedical Pioneering Innovation Center, Center for Reproductive Medicine, Department of Obstetrics and Gynecology, Peking University Third Hospital, Beijing 100191, China; Academy for Advanced Interdisciplinary Studies, Peking University, Beijing 100871, China; Beijing Advanced Innovation Center for Genomics (ICG), Ministry of Education Key Laboratory of Cell Proliferation and Differentiation, Beijing 100871, China; Peking-Tsinghua Center for Life Sciences, Academy for Advanced Interdisciplinary Studies, Peking University, Beijing 100871, China

**Keywords:** Human spermatogenesis, Non-obstructive azoospermia, Chromatin accessibility, Double-strand breaks, Transcription factor regulatory network

## Abstract

Spermatogenesis is an intricately regulated epigenetic process, yet the exact chromatin dynamics during human meiosis and their systemic failure in non-obstructive azoospermia remain elusive due to the sparsity of single-cell epigenomic data in previous studies. Here, leveraging a high-quality scATAC-seq approach, we mapped over 810,000 *cis*-regulatory elements across human spermatogenesis. We uncovered a highly synchronized, wave-like activation of master transcription factor networks that precisely orchestrates meiotic progression. Crucially, we traced the dynamic process of opening and closing of chromatin states at meiotic DNA double-strand break hotspot regions at single-cell resolution, defining a transient epigenetic window where thousands of these predefined regions become specifically accessible. In NOA patients, this delicate architecture collapses, leading to severe spermatogenic arrest predominantly at the zygotene stage. We characterize this pathogenesis as the temporal dephasing and decoupling of the activities of master TFs. Furthermore, aberrantly reduced chromatin accessibility at DSB hotspot regions in NOA is potentially associated with defective DSB formation, accompanied by a two- to three-fold increase in sperm aneuploidy. Finally, we establish a functional paradigm for interpreting male infertility by linking non-coding GWAS variants (e.g., the MHC II locus) and rare point mutations to the disruption of *cis*-regulatory elements. Together, our findings establish a high-resolution epigenetic framework for human spermatogenesis and provide a mechanistic basis for decoding the etiology and heterogeneity of non-obstructive azoospermia.

## Introduction

Spermatogenesis is a complex and highly coordinated developmental process where spermatogonial stem cells undergo multiple cell fate transitions to ultimately form morphologically and functionally mature sperm (Chen et al., 2018; Guo et al., 2018; Huang et al., 2023; Sohni et al., 2019; Wang et al., 2018). Within this continuous trajectory, the first meiotic division represents one of the most intricate and error-prone stages, involving critical biological events such as the formation and repair of meiotic DNA double-strand breaks (DSBs), synaptonemal complex assembly, and homologous recombination (Baudat et al., 2013).

The precise activation and subsequent appropriate repression of transcription factors (TFs) are fundamental to tightly regulating this extremely intricate biological process. Various master TFs must be activated at highly specific stages to initiate the expression of genes essential for male germ cell development. Once these genes fulfill their roles, the corresponding TFs must be properly downregulated to prevent prolonged expression, which could otherwise disrupt further cellular differentiation. This highly synchronized, wave-like regulatory balance ensures the orderly progression of meiosis, chromatin remodeling, and spermatid maturation, ultimately ensuring the production of functional sperm.

Genetic mutations or epigenetic variations can easily disrupt this delicate regulatory balance, leading to male infertility (Handel and Schimenti, 2010; Hu et al., 2011, 2014; Zhao et al., 2012), with non-obstructive azoospermia (NOA) representing one of the most severe clinical forms (Cerván-Martín et al., 2022; Hu et al., 2011, 2014; Kanai et al., 2018). While some NOA cases are attributed to chromosomal abnormalities—such as Klinefelter syndrome (KS), Y chromosome azoospermia factor (AZF) microdeletions, or Sertoli cell-only syndrome (SCOS)—over 70% of cases are classified as idiopathic NOA (iNOA) (Dabaja and Schlegel, 2013; Hamada et al., 2013). Although large-scale genome-wide association studies (GWAS) have linked specific genetic variants, such as those in the MHC II region, to male infertility (Cerván-Martín et al., 2022; Hu et al., 2011, 2014; Kanai et al., 2018), the specific molecular and epigenetic consequences of these variants on meiotic arrest remain largely unexplained.

Previous studies have successfully mapped the transcriptional and DNA methylation landscapes of spermatogenesis (Guo et al., 2018; Sohni et al., 2019; Wang et al., 2018), and have begun to explore chromatin accessibility dynamics (Wang et al., 2024, 2025; Wu et al., 2022). However, these investigations are often limited by small sample sizes, the averaging effect of bulk sequencing, or the sparsity of low-quality single-cell ATAC-seq data. These limitations have hindered a comprehensive, high-resolution understanding of the highly heterogeneous epigenetic events—such as the transient chromatin accessibility dynamics at DSB hotspot regions—during the first meiotic division. Consequently, research of the epigenetic dysregulation driving spermatogenic arrest in NOA patients remains severely restricted.

To bridge this gap, this study employed a newly developed, high-quality scATAC-seq technology (Liu et al., 2024) that provides unprecedented temporal resolution and fragment capture rates. We integrated this approach with transcriptomic and ChIP-seq data to systematically investigate the single-cell epigenetic features of spermatogenesis in 16 obstructive azoospermia (OA) patients, where spermatogenesis proceeds normally, and 15 NOA patients (encompassing iNOA, KS, AZFc microdeletion, and SCOS subtypes). Through this comprehensive high-quality atlas, we aimed to uncover the dynamic epigenetic regulatory mechanisms driving normal meiosis and to define how their disruption leads to defective spermatogenesis (Fig. 1A).

**Figure 1.**
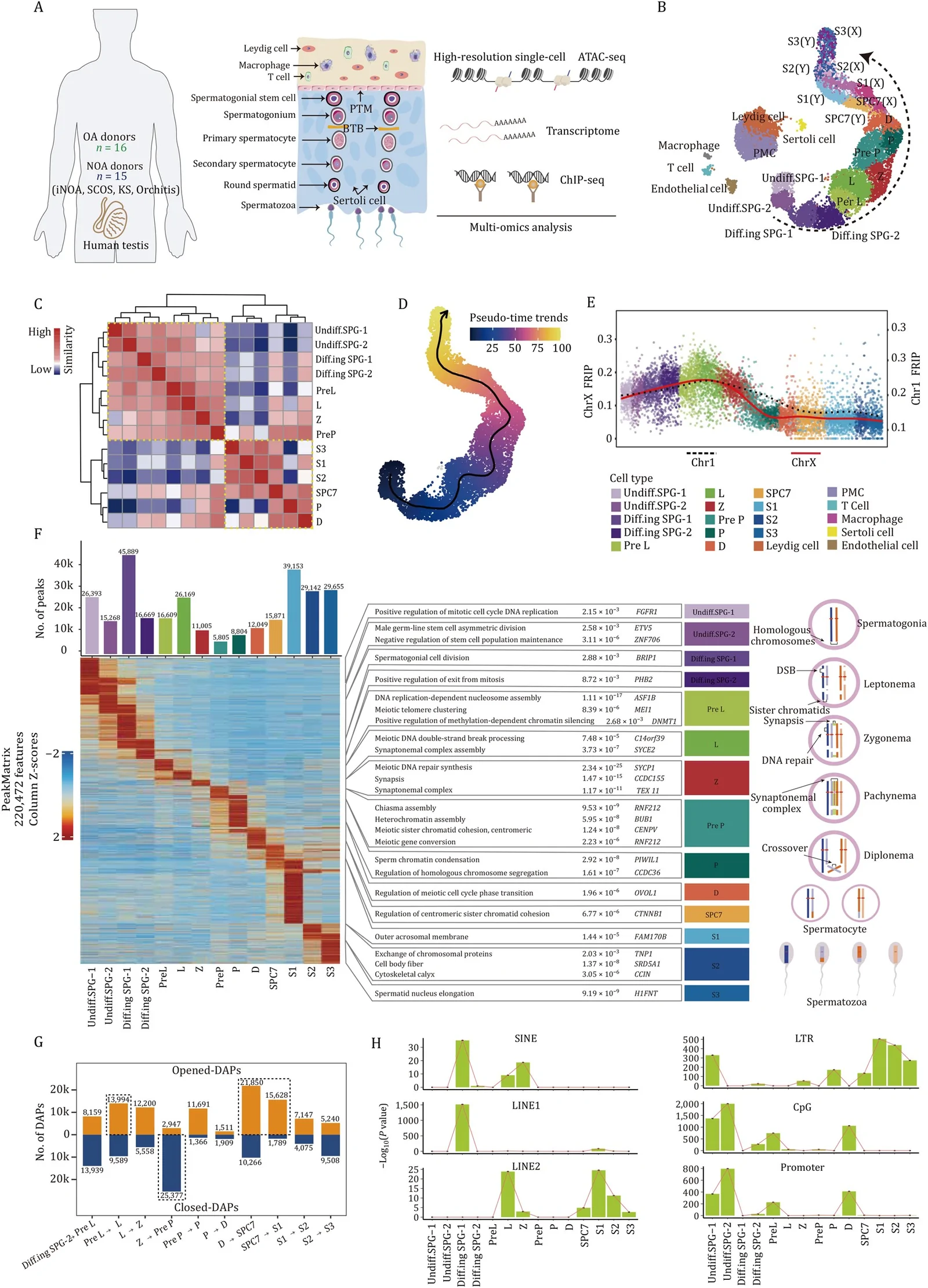
**Chromatin accessibility atlas of adult human spermatogenesis**. (A) Overview of the study design, including sample collection, schematic of cellular diversity during human spermatogenesis, and data analysis workflow. (B) Uniform manifold approximation and projection (UMAP) embedding of scATAC-seq data from 16 OA samples after iterative LSI dimensionality reduction. Each dot represents a single cell (*n* = 12,438), colored by annotated clusters. (C) Heatmap showing pairwise similarity and hierarchical clustering across consecutive spermatogenic stages. (D) Pseudotime trajectory from SPG to spermatids in OA cells. (E) Fraction of reads in peaks (FRiP) values for chromosome 1 and chromosome X along the pseudotime trajectory. Dots represent the FRiP values for chromosome X. (F) Left: Heatmap depicting cell type-specific chromatin accessibility peaks (*n* = 220,472), accompanied by their associated Gene Ontology (GO) terms (analyzed via GREAT) and representative genes. Right: Schematic illustration of chromosomal structural changes during spermatogenesis. (G) Bar plot showing the number of newly opened and closed differentially accessible peaks (DAPs) across consecutive developmental stages. (H) Enrichment of genomic and repetitive elements within cell-type-specific chromatin accessibility peaks.

## Results

### Chromatin accessibility profiling of adult human testicular cells

To obtain a high-quality chromatin accessibility atlas of human spermatogenesis, we utilized our previously developed scATAC-seq method (Liu et al., 2024) to profile testicular tissues from 16 patients with OA (Table S1–5). It is well-established that the spermatogenic process in OA patients is generally normal (Chen et al., 2023; Wang et al., 2018). Following FACS-based cell sorting and sequencing library preparation, we achieved high-quality data. Notably, when systematically compared to a publicly available human spermatogenesis scATAC-seq dataset generated using the standard 10x Genomics platform (Wang et al., 2025), our method demonstrated overwhelming advantages (Fig. S1A). Specifically, our raw dataset exhibited a significantly higher transcription start site enrichment (TSS enrichment) and captured vastly more fragments per cell, far exceeding the general metrics of the 10× platform. Consequently, when applying the stringent quality control thresholds defined in our study to both datasets, nearly 70% of the cells processed with our method successfully passed the QC, whereas only a negligible fraction (<5%) of the 10× dataset met these criteria. After this rigorous filtering, we retained 12,438 high-quality cells for subsequent analyses (Table S2 and Fig. S1B), identifying a total of 623,081 accessible peaks across the genome.

Through unsupervised cell clustering and evaluation of chromatin accessibility around well-known marker genes (gene activity scores) (Di Persio and Neuhaus, 2023; Huang et al., 2023; Wang et al., 2018), we successfully annotated 14 distinct types of male germ cells and 6 types of testicular somatic cells (Figs. 1B and S1F). Specifically, these include two developmental stages of undifferentiated spermatogonia (Undiff.SPG-1 and Undiff.SPG-2), which are characterized by high gene activity scores for *PIWIL4* and *UTF1*. Concurrently, elevated activity scores for *DMRT1* and *REC8* distinguish two subtypes of differentiating spermatogonia (Diff.ing SPG-1 and Diff.ing SPG-2). Primary spermatocytes were divided into six subpopulations (preleptotene (PreL), leptotene (L), zygotene (Z), pre-pachytene (PreP), pachytene (P), and diplotene (D)) based on gene activity scores like *DPH7*, *DMC1*, *SPO11*, *TEX19*, *SYCP1*, *TDRG1*, and *OVOL1* (Adams and Davies, 2023; Bhattacharyya et al., 2019; Gómez-H et al., 2016). Then, three spermatid subpopulations (S1, S2, S3) were identified based on *TXNDC2*, *PRM1*, and *PRM2* activities (Fig. S1C–E). Additionally, we captured a transitional cell type intermediate between spermatocytes and round spermatids, which we defined as secondary spermatocytes (SPC7). Furthermore, six testicular somatic cell types were also identified: Sertoli (*BEX1*), Leydig cells (*INSL3*), Peritubular myoid cells (PMC) (*DCN*), Macrophages (*CD14*), T cells (*CD3D*), and Endothelial cells (*PECAM1*) (Fig. S1C). Notably, gene activation patterns exhibit a higher degree of similarity from Undiff.SPG to Z, whereas they are more analogous from P to spermatids. The PreP stage is particularly distinct, serving as a transitional phase that marks a drastic turning point in the process of spermatogenesis (Fig. S1E and 1C). During the PreP period, chromatin in spermatocytes undergoes extensive condensation, followed by comprehensive chromatin remodeling.

To assess chromatin accessibility changes across autosomes and sex chromosomes during spermatogenesis, we constructed a semi-supervised pseudotemporal trajectory from the SPG to the spermatid stage (Fig. 1D). Along this trajectory, we calculated the chromosome-specific fraction of reads in peaks (FRiP) values for chromosome 1 and the X chromosome to track their epigenetic dynamics. We observed a global increase in chromatin compaction beginning at the PreP stage, which peaked at the D stage. Notably, the compaction of the X chromosome was more pronounced than that of chromosome 1, reaching its maximum extent by the P stage. This observation is consistent with meiotic sex chromosome inactivation (MSCI) (Turner, 2007) (Fig. 1E). Subsequently, following the first meiotic division, the X chromosome undergoes reactivation in post-meiotic spermatids, as evidenced by the activation scores of X-linked genes inferred through AUCell analysis (Aibar et al., 2017) (Fig. S1G).

By evaluating the ratio of reads mapping to chromosome Y versus chromosome X (chrY/chrX read ratio), we observed a distinct bimodal distribution, which enabled the robust distinction of secondary spermatocytes and haploid sperm cells carrying either the X or Y chromosome (Fig. S1H). Applying strict ratio thresholds (chrY/chrX < 0.05 for X-sperm and > 0.15 for Y-sperm), we confidently classified the haploid cells into two subgroups: 1,463 X-sperm cells and 1,530 Y-sperm cells.

### Dynamics of chromatin accessibility profiles during human spermatogenesis

To better understand the dynamics of chromatin accessibility alterations across different stages of human spermatogenesis, we identified marker peaks for each of the 14 germ cell types (Wilcoxon false discovery rate [FDR] ≤ 0.05, log_2_(fold change [FC]) ≥ 1; Fig. 1F). The number of marker peaks varied, ranging from 45,889 (Diff.ing SPG-1) to 5,805 (PreP). Subsequently, we performed functional enrichment analysis on these peaks (Fig. 1F). In the Undiff.SPG stage, cells proliferate and differentiate, undergoing mitosis. As they transition to the Diff.ing SPG stage, cells gradually exit mitosis. In the PreL stage, increased chromatin accessibility around *ASF1B* and *DNMT1* suggests the initiation of DNA replication again (Moiseeva and Bakkenist, 2018), accompanied by DNA demethylation (Huang et al., 2023). At the L stage, chromatin accessibility around *SYCE2* gradually increases, indicating synapse formation (Adams and Davies, 2023), with *C14orf39* activation leading to DNA DSB and the formation of recombination hotspots. By the Z stage, the DNA DSBs are gradually repaired (Bhattacharyya et al., 2019; Lingg et al., 2022). In the PreP stage, chromatin condenses globally (Prakash et al., 2015), accompanied by sex chromosome inactivation and sister chromatid cohesion, preparing for homologous recombination. GO enrichment analysis reveals a conversion in meiotic gene expression during the PreP stage, highlighting PreP as a transitional phase. During the late P and D stages, homologous chromosomes separate and meiotic cells divide. By the SPC7 stage, changes in sister chromatid cohesion indicate their impending separation. Finally, at the spermatid stage, sperm cells transition from a round to an elongated shape as the sperm nucleus gradually extends.

### Three key periods of chromatin accessibility changes during meiosis of spermatocytes

At each stage of human spermatogenesis, chromatin accessibility undergoes multiple dynamic changes, particularly upon entering meiosis. By comparing differential accessibility peaks (DAPs) between adjacent stages (FDR ≤ 0.05, log_2_FC ≥ 1), we identified three periods of significant chromatin accessibility changes: 1. from the PreL to L stage; 2. from the Z to PreP stage; 3. during the transition of spermatocytes to round spermatids (Fig. 1G).

Firstly, during the transition from the PreL to L stage, a total of 13,994 genomic regions showed increased accessibility (Fig. 1G), previously reported to be associated with PRDM9 activation and the formation of DSB hotspots (Chen et al., 2020; Lam et al., 2019) (Fig. S2A). Additionally, we observed 26,169 specific accessible peaks during the L stage (Fig. 1F), which significantly enriched for transposable elements SINE and LINE2 (Fig. 1H), with particularly notable enrichment in the Alu subfamily of SINE (Fig. S1I). This suggests that several key events during this period are more likely connected to the activation of these transposable elements (Johnson et al., 2013; Spangenberg et al., 2011). Previous research has shown significant enrichment of LINE2 and Alu elements in recombination hotspot regions (Myers et al., 2008). To distinguish the functions of these two elements, we integrated human male DMC1 ChIP-seq data, which covers 62,038 genomic regions representing DNA DSB sites involved in meiotic homologous recombination (Pratto et al., 2014). We found that chromatin accessible regions overlapping with PreL to L stages and DMC1-binding regions were highly enriched in LINE2 (Fig. S2B). However, when excluding DMC1-binding regions, Alu elements became more enriched (Fig. S2B). Our findings, alongside recent multi-omics studies of human sperm (Huang et al., 2023), suggest that LINE2 is more likely to be enriched in recombination hotspot regions compared to Alu. The enrichment of Alu may be correlated with the formation of chromosomal loops and the synaptonemal complexes (Johnson et al., 2013).

The second wave occurs during the transition from the Z to PreP stage, where 25,377 chromatin regions decreased accessibilities (Fig. 1G). This closure is anticipated and is associated with sex chromosome inactivation and global chromatin compaction. Functional enrichment analysis suggests that the genes neighboring these chromatin regions with decreased accessibility are significantly enriched for annotations related to H3K27 modifications (Prakash et al., 2015) (Fig. S2A).

The final wave occurs during the transition of spermatocytes to round spermatids, where chromatin accessibility continuously increases across two developmental stages (Fig. 1G). Functional enrichment analysis links this global peak opening to the exit from meiosis and round spermatid formation (Fig. S2A). In the S1 stage, 39,153 specific open chromatin peaks (Fig. 1F) were significantly enriched in transposable element LTRs (Fig. 1H). Previous studies have suggested that the enrichment of LTRs in round spermatids may be associated with increased transcriptional activity of lncRNAs (Davis et al., 2017). LTRs can act as alternative promoters for some genes (Flemr et al., 2013), and their activation in S1 may drive the transcription of nearby genes, particularly lncRNAs. Expression analysis shows the highest lncRNA expression in the S1 stage (Wang et al., 2018) (Fig. S2C). We identified 2,460 upregulated genes in S1 compared to SPC7 (*P-*value ≤ 0.05, log_2_FC ≥ 0.5), 723 of which contain LTRs in their promoter regions. Among these, 90 are lncRNAs (Fisher’s exact test, *P *= 0.003) (Fig. S2D and 2E), suggesting that LTR enrichment in open chromatin peaks during S1 is linked to lncRNA activation. Two examples illustrate this: *LINC00558*, an lncRNA on chromosome 13, has two LTR copies in its promoter and gene body. In the SPC7 stage, these LTRs lack accessibility, and *LINC00558* is not transcribed. However, in the S1 stage, the LTRs become accessible, leading to increased transcription of *LINC00558* (Fig. S2F). Similarly, the *SUN5* gene, a round spermatid marker with an LTR in its promoter, shows increased accessibility in S1, resulting in a significant rise in *SUN5* expression levels (Fig. S2G).

### Master transcription factors involved in human spermatogenesis showed synchronized and wave-like activities

The fate and state of each cell are orchestrated in part through complex circuits of TFs, converging at regulatory elements enabling precise control of gene expression (Fleck et al., 2023). TF deviation scores, as computed by chromVAR (Granja et al., 2021; Schep et al., 2017), offer insights into the activity levels of multiple TFs sharing similar DNA-binding motifs (Schep et al., 2017). We utilized these scores to identify the top-ranking TFs exhibiting the greatest variability in motif activity scores across spermatogenesis, including DMRT1, NFYB, RFX2, CTCF, and YY1 (Fig. S3A). These TFs showed significant motif activation differences among germ cell types, suggesting their potential pivotal roles in orchestrating epigenetic regulation transitions during male germ cell development. Subsequently, we calculated the cell-type-specific TF with higher motif activity, where “MeanDiff” represents the specificity of the TF (Fig. S3B).

To investigate the dynamics of TF motif activities during human spermatogenesis, we correlated TF motif activities with gene activities along the spermatogenesis trajectory to identify positive TF regulators and potential driver TFs of human spermatogenesis (Fig. 2A). For example, we observed that at the SPG stage, the GATA3 motif activities and gene activities are relatively high. In early-spermatocytes, CTCF, a major regulator of chromatin three-dimensional structures, become activated, preparing for the construction of specific chromatin structures for the first meiotic division, while the NFY family also exhibit high activities. YY1 shows high activities throughout spermatocytes. Additionally, YBX1, USF2, and USF1 potentially play important roles in the late-spermatocytes. RFX family exhibits high activation at the spermatid stage.

**Figure 2.**
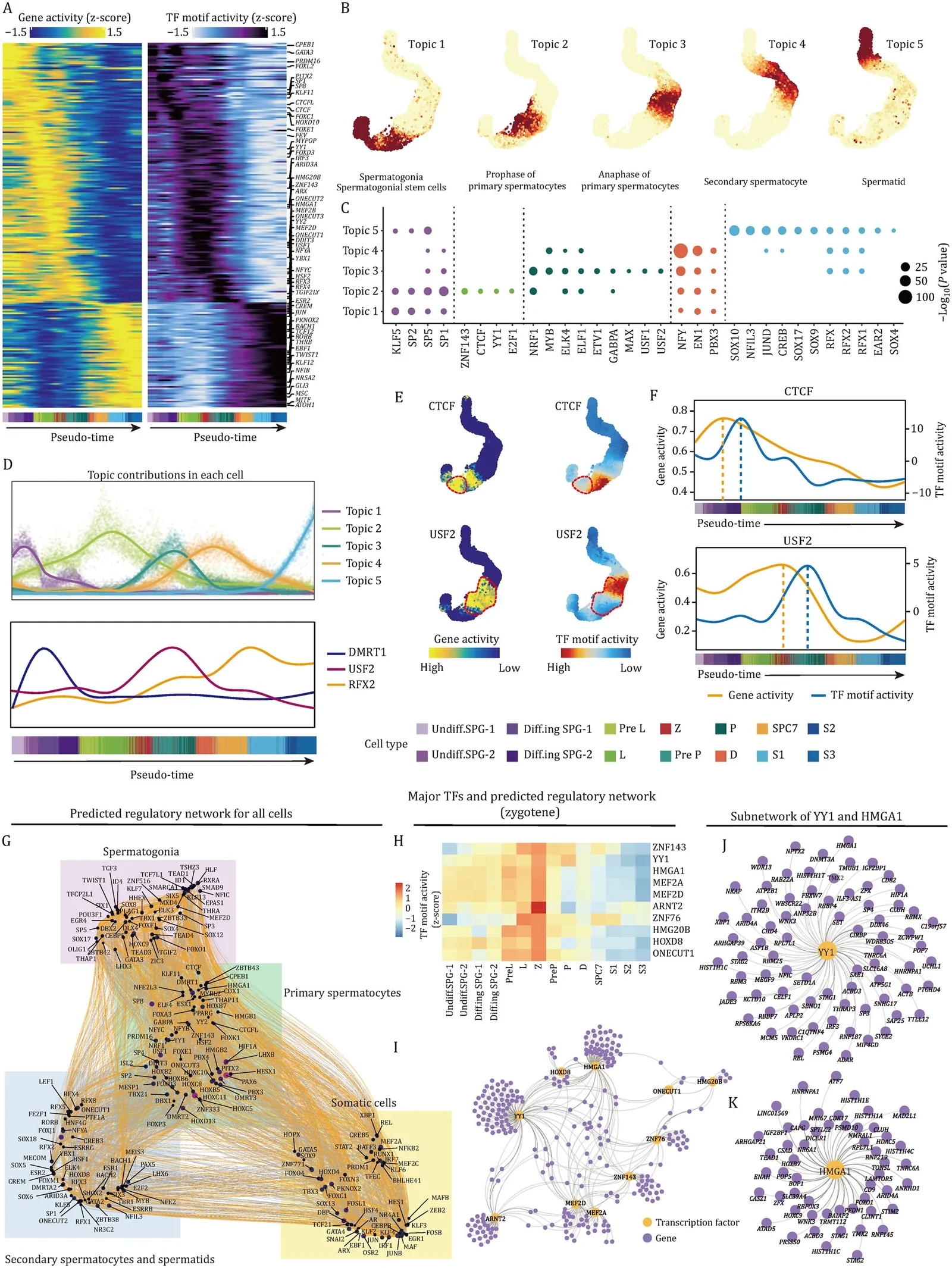
**Construction of gene regulatory network in human spermatogenesis**. (A) Heatmap showing TFs that exhibit a high correlation between their gene activity scores and motif activity scores along the trajectory from SPG to spermatids. (B) UMAP plots illustrating topics 1–5, which correspond to various stages of spermatogenesis. (C) Motif enrichment for a selected subset of topics. Dot size represents the significance of enrichment (identified via HOMER). (D) Top: Trend lines representing the contribution of five topics across individual cells, ordered along the pseudotime trajectory. Bottom: Illustrative examples of motif activation score trends along the same trajectory for DMRT1 (associated with Topic 1), USF2 (Topic 3), and RFX2 (Topic 5). (E) UMAP visualization of gene activity scores and corresponding TF motif activity scores. (F) Trends of CTCF and USF2 motif activity scores and gene activity scores along the pseudotime trajectory. (G) UMAP embedding of the inferred TF regulatory network based on co-expression and inferred interaction strengths between TFs. Node color and size represent the expression-weighted pseudotime and PageRank centrality of each TF, respectively. (H) Heatmap displaying the motif activity scores of zygotene spermatocyte-specific TFs. (I) Zygotene spermatocyte-specific TF network visualized using the ForceAtlas layout algorithm. Node distances represent the correlation strength. (J) Potential target genes positively regulated by YY1 during the zygotene (Z) stage. (K) Potential target genes positively regulated by HMGA1 during the Z stage.

Next, we used cisTopic (Bravo González-Blas et al., 2023) to identify accessible region topics, which represent groups of accessible regions that covary and are likely to represent related biological programs. Based on the point where the log-likelihood estimation and topic coherence scores reached saturation, we fitted a model with 14 topics (Fig. S3C), 5 of which are related to germ cells (Fig. 2B). Topics 1–5 correspond to the five stages of spermatogenesis, with each topic enriching for key transcription factors specific to its stage (Figs. 2C and S3D). For instance, topic 2, which represents early meiotic prophase, is enriched for motifs of CTCF, ZNF143, and YY1 (Fig. 2C).

Most importantly, we found that distinct topics and their enriched master TFs exhibit wave-like patterns of activation and repression. Additionally, different master TFs within the same topic demonstrated highly synchronized activities (Fig. 2D). These highly coordinated and wave-like dynamics resemble a regulatory relay, where specific master TFs with coupling effects are activated at precise developmental stages in an ascending wave to initiate key biological processes. Once these functions are completed, the waves recede as these critical TFs are synchronously repressed, allowing the subsequent wave of a different set of master TFs to take over, ensuring the highly ordered progression of spermatogenesis.

Furthermore, we observed that gene activities of some TFs increase much earlier than their motif activities. For instance, although the motif activity of USF2 peaks in late-stage spermatocytes, its gene activity reaches its maximum in early-stage spermatocytes already (Fig. 2E). Similar patterns are observed for genes like *CTCF* and *RFX2*. This phenomenon indicates that for a transcription factor to function, an increase in chromatin accessibility of its promoter region is necessary to enhance gene transcription. Subsequently, the produced TF proteins will activate their target genes until much later developmental stages.

### Construction of TF regulatory networks for human spermatogenesis

To construct the gene regulatory network (GRN) of human spermatogenesis, we integrated high-quality single-cell ATAC-seq data with single-cell RNA-seq data. For the scRNA-seq component, we incorporated published single-cell transcriptomic data generated from patients matching the same hospital and department, thereby ensuring high consistency in clinical diagnostic criteria and sample oversight (Wang et al., 2018). Firstly, we constructed “simulated multi-omic metacells” containing information on both transcriptome and chromatin accessibility using Seurat (Stuart et al., 2019), and the integrated data were visualized using UMAP embedding (Fig. S3E). The cell types obtained from scATAC-seq correspond well with those obtained from scRNA-seq (Fig. S3F).

Next, we constructed the gene regulatory network of human spermatogenesis using Pando (Fleck et al., 2023) (Table S3), which identifies TF clusters at different stages, broadly reflecting the pseudotemporal order of cell state transitions. Subsequently, we visualized this network at the TF level using UMAP embedding (Fig. 2G). The visualization unveiled distinct TF groups activated in spermatogonia (ID4, POU3F1, FOXO1, EGR4, KLF7, GATA3), primary spermatocytes (CTCF, HMGA1, ZNF143, YY1, FOXD3, PITX2, DMRT1), secondary spermatocytes and spermatids (RFX2, CREM, FOXJ1, HSF1, SOX5, SOX6), as well as regulatory interactions between the TFs.

To infer cell-type-specific regulatory networks, we filtered the global GRN based on cell type chromatin accessibility and TF motif activity calculated by chromVAR. At the Z stage, we focused on TFs with high motif activities, such as ZNF143, YY1, HOXD8, and HMGA1, to construct the Z stage subnetwork, including both positive and negative regulatory interactions (Fig. 2H and 2I). YY1 and HMGA1 were identified as central nodes with numerous positive regulatory connections (Fig. 2J and 2K).

Notably, *HIST1H1T*, a testis-specific linker histone variant involved in meiotic chromatin remodeling (MacHida et al., 2016), was predicted as a key target gene of YY1 during this stage (Fig. S3G). Similarly, HMGA1-targeted genes such as *STAG1*, a core subunit of the cohesin complex providing the foundational structural framework for synaptonemal complex assembly during the zygotene stage (Ishiguro, 2019), as well as *ATAD5*, which facilitates DNA DSB repair and homologous recombination (Park et al., 2023), were also prominent (Fig. S3H). In summary, constructing this regulatory network helps us understand the dynamic epigenetic regulation process of spermatogenesis.

### The dynamics of chromatin accessibility at meiotic DNA DSB hotspots during human spermatogenesis

Meiotic recombination is essential for accurate chromosome separation and the generation of genetic diversity (Hunter, 2015). During the L and Z stages of human spermatogenesis, PRDM9 recruits SPO11 to induce DNA DSBs at specific recombination hotspots (Bhattacharyya et al., 2019; Pratto et al., 2014). These DSBs are subsequently repaired via homologous recombination involving DMC1, leading to crossovers or non-crossovers (Fig. 3A). To explore the epigenetic licensing of these events, we integrated DMC1-defined DSB hotspot data (Pratto et al., 2014) with our high-resolution scATAC-seq atlas. UMAP visualization demonstrated that chromatin accessibility at these hotspots peaks precisely during the L and Z stages (Fig. 3B). Specifically, 23.1% of the 141,661 total peaks at the L stage overlapped with DMC1 hotspots, compared to a baseline of ∼6% in non-meiotic cells (Fig. S4A). The signal intensity around these hotspot centers peaked at the Z stage (Fig. 3C), revealing a transient accessible window of approximately 265 bp in width (Fig. S4B).

**Figure 3.**
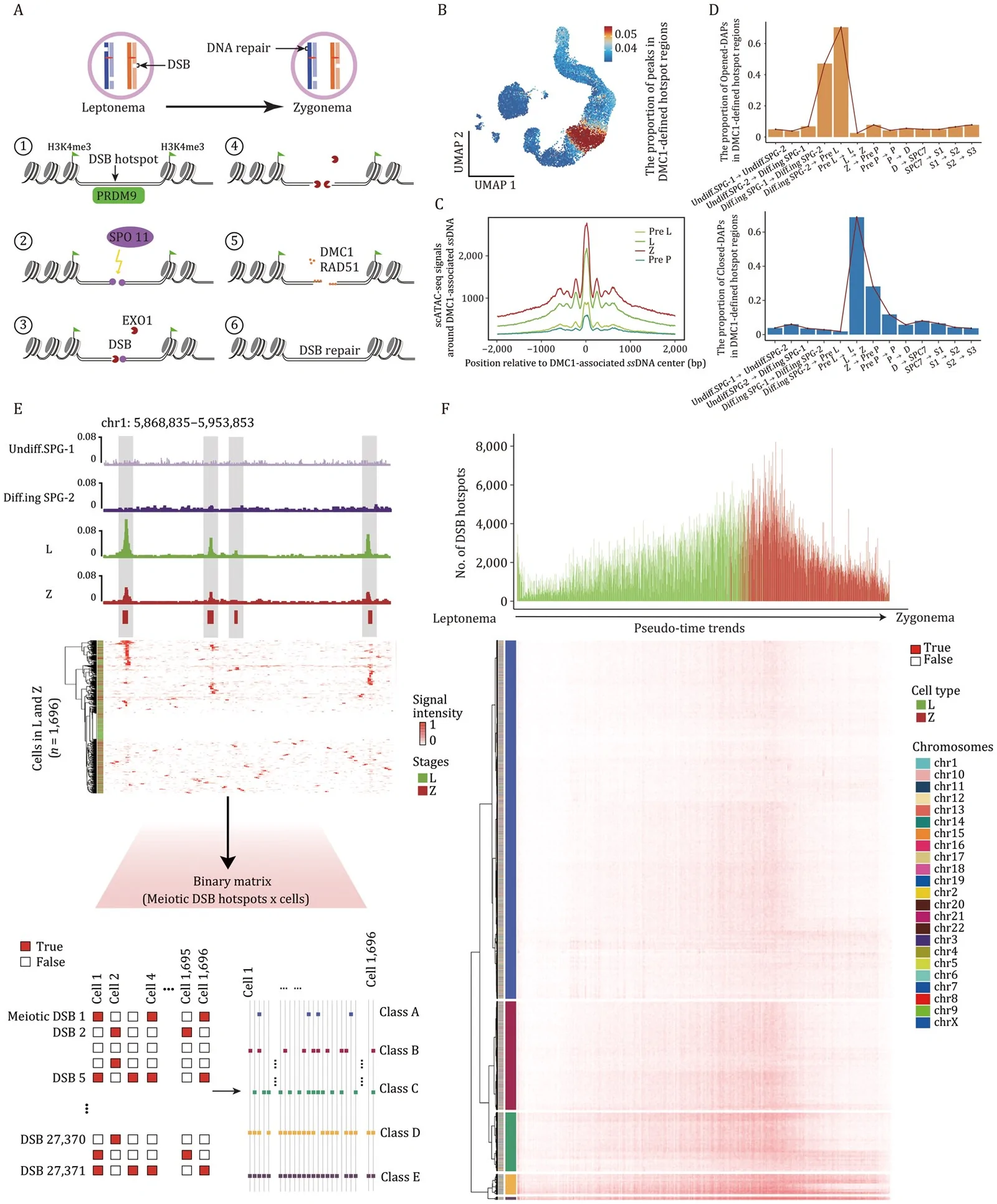
**Dynamics of chromatin accessibility at meiotic DNA DSB hotspots in human male germ cells**. (A) Schematic representation of meiotic DNA DSB formation and repair processes during the leptotene (L) and zygotene (Z) stages of spermatogenesis. (B) UMAP visualization of the proportion of peaks overlapping with DMC1-defined hotspot regions. (C) ATAC-seq signals around DMC1-defined hotspot centers (±2 kb) during the PreL, L, Z, and PreP stages. (D) Bar plot showing the proportion of newly opened and closed DAPs within DMC1-defined hotspot regions across consecutive spermatogenic stages. (E) Overview of the single-cell binary matrix design for evaluating chromatin accessibility at meiotic DSB hotspots. (F) Single-cell binary matrix showing the dynamics of chromatin accessibility at meiotic DSB hotspots. The top bar plot indicates the number of accessible meiotic DSB hotspots covered by each cell. Left color bars indicate hotspot classes and their chromosomal distributions.

Rather than observing static snapshots, we focused on the dynamic process of opening and closing of chromatin accessibility at these regions across consecutive meiotic stages. By analyzing DAPs, we found that nearly 70% of the newly opened regions during the PreL to L transition overlapped with DMC1-defined hotspots. Subsequently, a similar proportion (∼70%) of the newly closed regions during the L to Z transition were also localized within these hotspots (Fig. 3D). To definitively determine if these gaining and losing events occurred at identical genomic loci, we intersected these two sets of hotspots. A Venn diagram analysis revealed a substantial and specific intersection: 48.0% (5,892/12,270) of the hotspots that newly gained accessibility during the Diff.ing SPG-2 to PreL & PreL to L transition corresponded exactly to 60.0% (5,892/9,817) of the regions that subsequently lost accessibility during the L to Z & Z to PreP transition (Fig. S4C). This strong overlap demonstrates that a major, continuous subset of DSB hotspots undergoes a sequential, highly dynamic process of rapid chromatin opening followed later by closing.

Crucially, the exceptionally high fragment capture rate of our method allowed us to reconstruct this process as a continuous developmental continuum. When investigating representative DMC1-defined hotspots, notable cellular heterogeneity was observed. To fully illustrate the distribution and continuous dynamics of DSB hotspot priming across individual male germ cells, we constructed a high-fidelity binary accessibility matrix (Fig. 3E). After stringent filtering to exclude promoter-proximal and Y-chromosome confounders (see “Methods” section for details), the final matrix comprised 27,371 refined DSB hotspots across 1,696 individual leptotene and zygotene germ cells. This extensive matrix allowed us to track the stochastic yet sequential opening of these regions. Along the reconstructed pseudotemporal trajectory from PreL to PreP (Fig. S4D), we observed a gradual and uninterrupted accumulation of accessible DSB hotspots starting from the L stage, reaching a peak at the Z stage (median ∼5,000 open hotspots per cell), followed by a coordinated resolution (Fig. 3F). This high-resolution trajectory reveals that while several thousand hotspots are epigenetically “primed” (likely via PRDM9 binding and H3K4me3 modification), only a subset (several hundreds) proceeds to form actual DSBs (Baudat et al., 2013), and ultimately, only a few dozen culminate in crossovers (Bell et al., 2020; Lu et al., 2012). To our knowledge, this represents the first observation of the complete, continuous dynamic process of chromatin accessibility changes at meiotic DSB hotspots along the human meiotic trajectory at single-cell resolution.

Next, unsupervised clustering of the binary accessibility matrix categorized the 27,371 meiotic DSB hotspots into five distinct classes (A, B, C, D, and E) (Fig. 3F). The median number of germ cells harboring these accessible hotspots increased progressively from 100 (5.9%) in class A to 925 (54.5%) in class E (Figs. 3F and S4E), reflecting a distinct gradient in their frequency of usage across the meiotic population. Furthermore, the distribution of these five hotspot classes exhibited no obvious chromosomal bias (Fig. S4F and Table S4).

To further elucidate their genomic characteristics, we conducted a genomic feature enrichment analysis (Fig. S4G). The results revealed that all five classes were enriched for LINE2 retrotransposons to varying degrees, highlighting a close structural or regulatory relationship between LINE2 elements and meiotic DSB formation. Additionally, these hotspots demonstrated a pronounced enrichment within sub-telomeric regions. Given that crossovers—which represent a successfully repaired subset of DSB hotspots—are known to cluster near telomeres in both human and mouse sperm (Bell et al., 2020; Xie et al., 2023), our single-cell chromatin accessibility data provide direct epigenetic evidence that the initial priming of meiotic DSBs inherently favors these sub-telomeric regions.

### Spermatogenesis in iNOA patients are arrested at the zygotene stage

Next, we analyzed the epigenetic characteristics of spermatogenesis in non-obstructive azoospermia (NOA) patients. We collected a total of 15 patient samples (Table S1), including 8 cases of idiopathic NOA (iNOA), 1 case of orchitis, 3 cases diagnosed with AZFc microdeletions, 2 cases of Sertoli cell-only syndrome (SCOS), and 1 case of Klinefelter syndrome (KS). Following stringent quality control, we retained 15,546 high-quality cells for subsequent analyses (Table S2). The data quality is very high, with an average TSS enrichment of 10.7 and an average of 78,944 fragments per cell (Fig. S5A). Utilizing dimensionality reduction and clustering analysis on all cells from 16 OA patients and 15 NOA patients (Fig. 4A), we mapped the cell type labels from OA samples to neighboring NOA cells, identifying 14 classes of germ cells and 6 classes of testicular somatic cells (Fig. 4B and 4C). Additionally, we identified 812,648 peaks.

**Figure 4.**
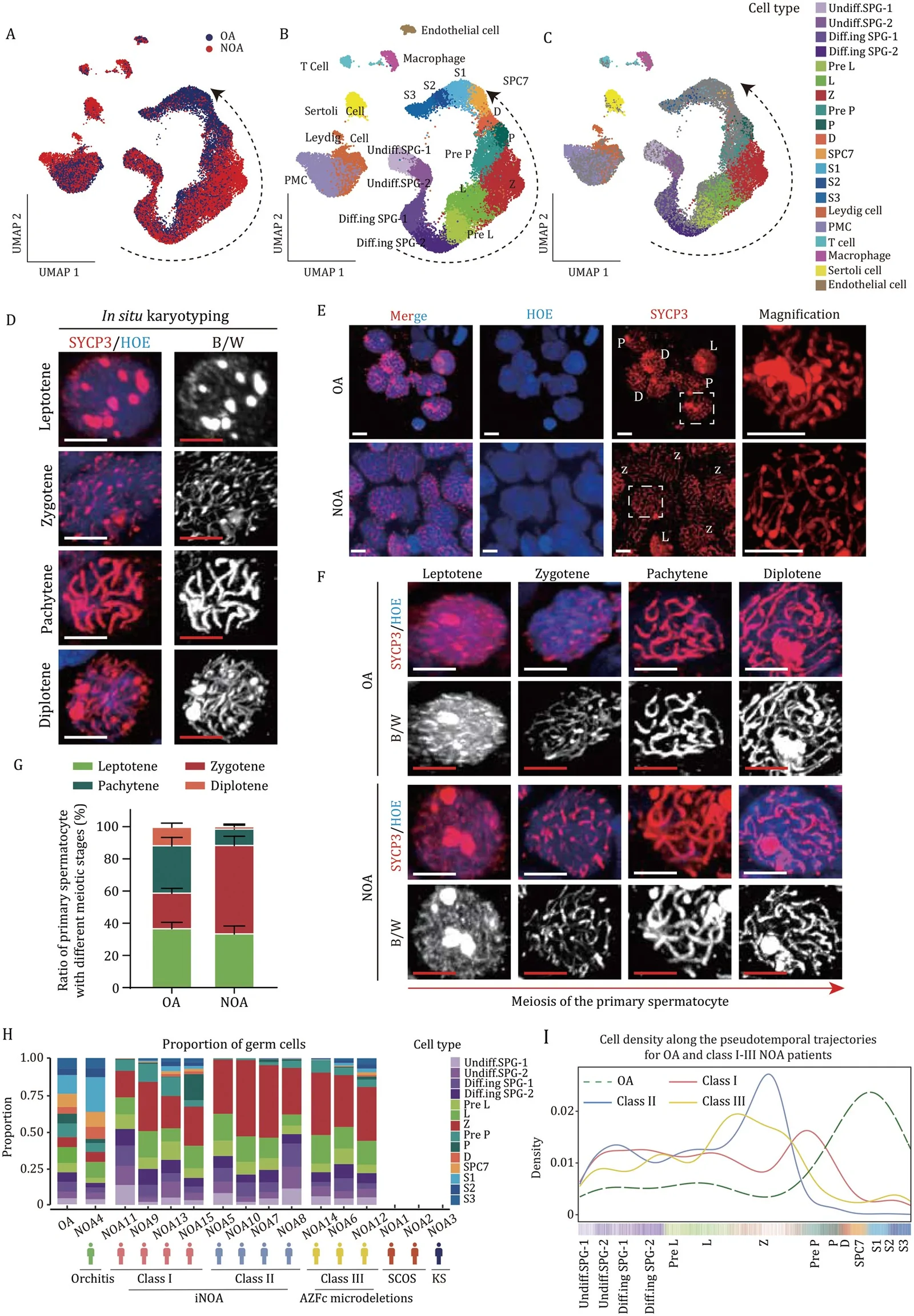
**Spermatogenic arrest at the zygotene stage in NOA patients**. (A) UMAP visualization of all single cells from 16 OA samples and 15 NOA samples, colored by sample type. (B) Same as in (A), but with each cell colored by cell type. (C) Same as in (A), but showing only cells from NOA samples, colored by cell type. (D) Representative *in situ* karyotyping images of the meiotic phases in embryonic germ cells. SYCP3, red; Hoechst (HOE), blue. B/W, the red fluorescence of SYCP3 was inverted to black/white (B/W) to highlight the meiotic stages in the right panel. (E) SYCP3 staining of human testes showed that primary spermatocytes of OA tissues were in different developmental stages, including the pachytene and diplotene; however, most germ cells of NOA patients were in the zygotene. (F) Co-immunostaining was performed using SYCP3 and HOE from adult human testis surface spreading. (G) Counting the ratio of leptotene, zygotene, pachytene, and diplotene germ cells in the testes of patients with OA and NOA (*n* = 3). All experiments were repeated at least three times, and representative images are shown. Data are presented as the mean ± SD and analyzed by a two-tailed unpaired Student’s *t*-test, ns, *P* ≥ 0.05 and ***P < 0.001. SYCP3, red; HOE, blue. Scale bars, 5 μm (D–G). (H) Cellular composition of germ cell clusters within each NOA sample. Total proportions for each germ cell cluster in OA are shown in the leftmost column. The 15 NOA patients are classified into 6 subclasses: Orchitis, Class I, Class II, Class III, SCOS, and KS. (I) Cell density along the pseudotime trajectories for OA and Class I–III NOA patients. The bar plot only shows the pseudotime trajectories from OA.

In adult males, seminiferous tubules contain layers of germ cells at progressive developmental stages. The failure of the first meiotic division in primary spermatocytes is a hallmark closely associated with NOA (Xie et al., 2022). To investigate this, we first utilized the meiotic chromosome marker SYCP3 (Dai et al., 2022; Prieto et al., 2004) coupled with a high-resolution confocal imaging system (Xu et al., 2022). This approach allowed us to clearly identify the meiotic phases—leptotene (L), zygotene (Z), pachytene (P), and diplotene (D)—in testicular sections (Fig. 4D). In OA patients, primary spermatocytes at continuous developmental stages, including the P and D stages, were radially distributed across the seminiferous tubules. In striking contrast, zygotene spermatocytes aberrantly clustered within the tubules of NOA patients (Fig. 4E). Statistical quantification confirmed that the majority of germ cells in NOA patients were arrested at the Z stage compared to the OA group (55.0 ± 5.3% vs. 22.1 ± 2.6%, Fig. 4F and 4G).

To robustly validate this meiotic arrest using independent approach, we assessed the dynamic spatial localization of γH2AX, a well-established marker for DSB repair and chromosome behavior. During male germ cell meiosis, the γH2AX staining pattern serves as a highly reliable criterion for staging primary spermatocytes. At the leptotene stage, γH2AX exhibits a diffuse distribution covering the entire nucleus, reflecting the abundant formation of DSBs as chromosomes begin to condense (Fig. S6A). At the Z stage, signals gradually concentrate along the pairing regions of homologous chromosomes, forming punctate or linear aggregates. At the P stage, γH2AX staining becomes distinctly restricted to the sex chromosome region (XY body), with autosomal signals significantly reduced. Finally, at the D stage, the signals are further diminished, leaving only a few residual puncta on certain autosomes (Fig. S6B).

Applying this rigorous γH2AX staging criterion, we consistently observed the normal radial distribution of progressively staging primary spermatocytes in the seminiferous tubules of OA patients. Conversely, the seminiferous tubules of NOA patients exhibited a pronounced abnormal clustering of leptotene and zygotene cells (Fig. S6B). Statistical analysis of the γH2AX signals further corroborated a significant increase in the proportion of these early meiotic germ cells in NOA compared to OA patients (Fig. S6C). Together, these dual-marker assessments definitively demonstrate that primary spermatocytes in NOA patients undergo an abnormal developmental arrest predominantly at the early meiotic stages.

By quantifying the number of each type of germ cell identified in every NOA patient, we also observed a significant accumulation of cells at the Z stage in the scATAC-seq data for most patients (Fig. 4H), accompanied by a minimal proportion of cells beyond this stage, ranging from 63% in NOA4 to 0.9% in NOA5 (Figs. S5B and 5C). This suggests that the spermatogenesis process in NOA patients was mainly arrested at the zygotene stage. The proportion of germ cells in NOA4 (orchitis) is similar to that in OA, indicating no developmental arrest, while SCOS and KS patients exhibited a complete absence of germ cells. By quantifying the degree of cell arrest at the Z stage (proportion of cells after the Z stage) for each patient, we classified patients into 6 groups, ranging from no arrest to severe arrest or absence of germ cells: NOA4 defined as Orchitis; NOA 9, 11, 13, and 15 defined as Class I; NOA5, 7, 8, 10 defined as Class II; NOA6, 12, 14 defined as Class III (AZFc microdeletions); NOA1, 2 defined as SCOS; NOA3 defined as KS (Fig. 4H and 4I).

### Abnormal synaptonemal complex formation in spermatocytes of NOA patients

To investigate the causes of spermatogenesis arrest in NOA patients, we separately counted the DAPs between each NOA class and OA across all cell types (FDR < 0.05 and |Log_2_FC| ≥ 0.5) (Fig. 5A). The results revealed a higher number of DAPs in Z spermatocytes and PMCs, followed by those in the L stage. Specifically, during the Z stage, Class I, II, and III exhibited 617, 6,853, and 13,907 specifically closed DAPs, respectively. Moreover, we performed GO enrichment analysis on these DAPs. We found that aberrantly closed DAPs in Class I, II, and III were significantly enriched in terms related to “meiotic DNA repair synthesis”, “lateral element assembly” and “chromosome organization involved in meiotic cell cycle” (Fig. 5B–D), while Class III was specifically enriched in terms related to synaptic formation (Fig. 5D). To further validate the enrichment analysis, we calculated the gene activity score of key genes involved in these GO terms (Fig. S7A). For instance, *SYCP1* is closely associated with synaptonemal complex formation (De Vries et al., 2005), *BUB1* is involved in meiotic sister chromatid cohesion (Marchetti and Venkatachalam, 2010), and *ACTR2* is linked to the meiotic cell cycle process and DNA DSB repair (Schrank et al., 2018). They were downregulated in all three classes of NOA patients. Meanwhile, genes related to prostate hormones, such as *PTGES3*, were downregulated in Class II and III, while genes related to synaptic formation, such as *CCDC155* and *CCDC36* (Ozturk, 2023), were downregulated in Class III (Fig. 5E).

**Figure 5.**
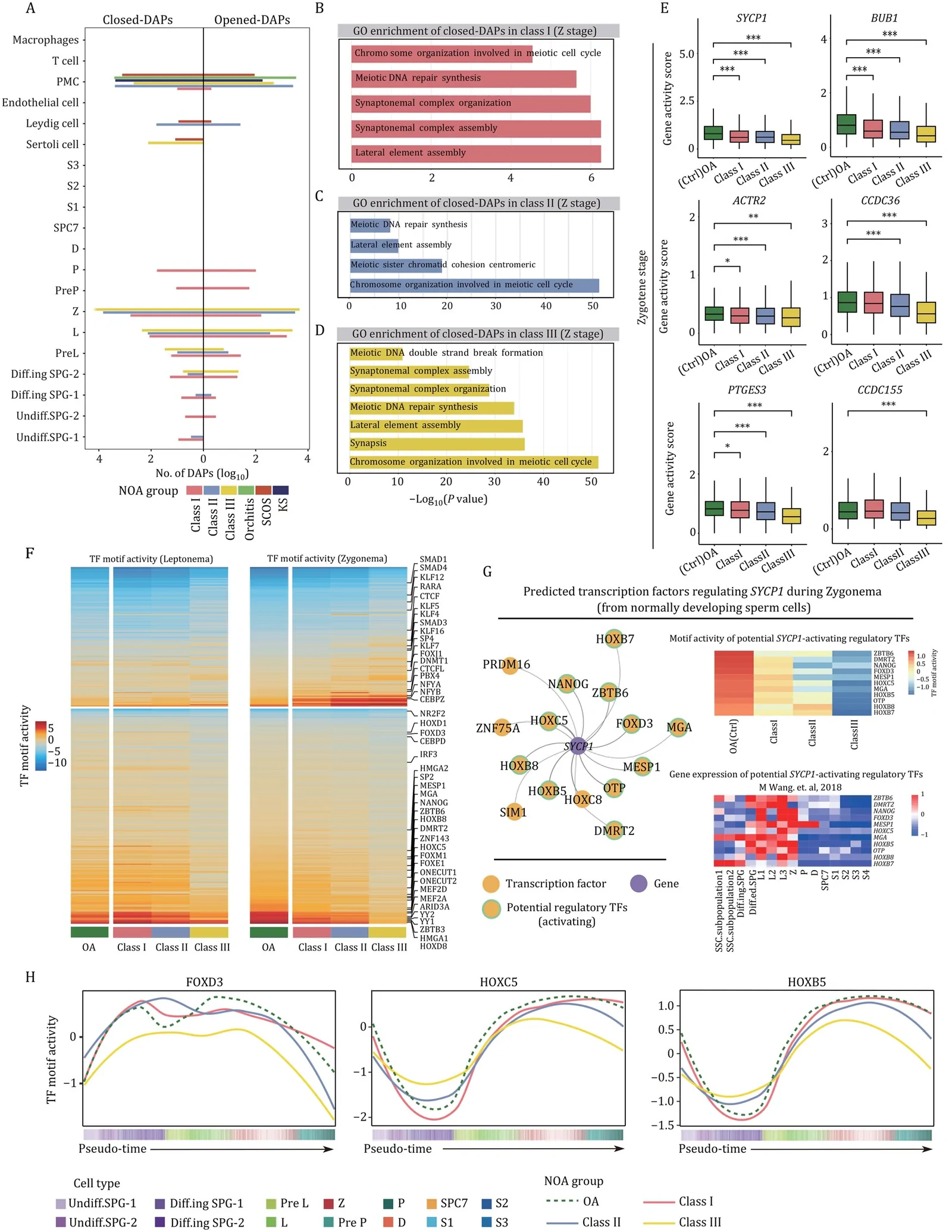
**Disrupted TF motif activation in zygotene spermatocytes of NOA patients**. (A) Bar plot showing the number of opened and closed DAPs between each NOA class and OA across all cell types. (B) Functional enrichment of GO biological processes for closed DAPs in Class I patients during the Z stage. (C) Functional enrichment of GO biological processes for closed DAPs in Class II patients during the Z stage. (D) Functional enrichment of GO biological processes for closed DAPs in Class III patients during the Z stage. (E) Comparison of gene activity scores between OA and Class I–III patients (Wilcoxon test, *P < 0.05, *P* < 0.01, ***P < 0.001). (F) Heatmap depicting differential TF motif activity between Class I–III patients and OA at the L and Z stages. The top section displays TFs with increased activity in NOA patients, while the bottom section shows TFs with decreased activity. (G) Predicted transcription factors regulating *SYCP1* during the Z stage (inferred from normally developing germ cells). (H) Trends of motif activity scores for selected TFs in OA and Class I–III patients along the pseudotime trajectory.

### Dephasing and decoupling of activities of master TFs in zygotene spermatocytes of NOA patients

To investigate TF motif activity dysregulation in spermatogenesis of NOA patients, we compared TF motif activity between Class I–III patients and OA at the Z stage (FDR ≤ 0.001 and abs(MeanDiff) ≥ 0.5), and these differential TFs needed to be expressed in normal zygotene spermatocytes (TPM > 0.01). Ultimately, 116 TFs were identified with upregulation of motif activities in any of Class I, II, or III, while 179 TFs showed downregulation of motif activities (Fig. 5F).

To further investigate TF motif activity dynamics, we conducted a semi-supervised pseudotemporal trajectory analysis across all germ cells, revealing distinct TF trends along the trajectories for OA and Class I-III NOA patients. We found that inadequate activation of certain TFs at the right time potentially contributes to defects in spermatogenesis. Specifically, for TFs like YY1, FOXE1, HMGA1, and ZNF143, motif activity is typically elevated during the Z stage in normal spermatogenesis to maintain the expression of functional target genes. However, in NOA patients, especially those classified as Class III, these motifs were insufficiently activated during the Z stage (Fig. 5F). Moreover, the failure to repress certain TFs at the appropriate time also contributes to the developmental arrest. For example, the downstream binding sites of TFs from the KLF family and CTCF need to be gradually closed after the Z stage (Fig. S7B). However, in NOA patients, these repressions did not occur effectively, hindering spermatogenesis from progressing to the next developmental stage properly.

In summary, we observed that the activities of a subset of master TFs decoupled in NOA patients, where these TFs either fail to activate sufficiently or are not repressed properly at the correct time points in the developmental timeline. Furthermore, under normal conditions, the master TFs operate in a highly synchronized manner to drive spermatogenesis. However, when a key TF is out of phase, this tightly coupled regulatory network collapses, causing downstream target gene expressions and corresponding molecular pathways that depend on this TF to become dysregulated (known as decoupling). The cumulative effect of these dephasing and decoupling events ultimately results in spermatogenic arrest.

Next, we investigated whether these aberrantly activated TF motifs influence the gene activity scores of their target genes, thereby impacting downstream gene expression. Specifically, given the critical role of the synaptonemal complex during the Z stage, we sought to determine the upstream regulatory mechanisms driving the suppressed gene activity of *SYCP1* in NOA patients. To achieve this, we employed Pando to construct a highly stringent, locus-centric regulatory network specifically focused on the accessible chromatin regions immediately surrounding the *SYCP1* locus. We established a strict four-tier filtering pipeline. First, we required that the specific regulatory peaks predicted to be bound by the candidate TFs around *SYCP1* exhibit significantly decreased accessibility (closed DAPs) in NOA (Class III) patients, compared to normal controls. Second, to ensure an activating regulatory relationship, the candidate TF must possess a positive regulatory coefficient (>0.1) with *SYCP1* during normal spermatogenesis. A total of 15 TFs met these initial locus-specific criteria (Fig. 5G and 5H). Third, we stipulated that these candidate TFs must be actively expressed during the normal Z stage. Finally, we required that the global motif activity of these candidate TFs also be significantly downregulated in NOA patients.

By applying this comprehensive filter, we identified 11 high-confidence TFs whose coordinated failure is strongly implicated in *SYCP1* suppression. Furthermore, the majority of these TFs reach their peak gene expression around the Z stage (Fig. 5G). This temporal synchrony aligns perfectly with the developmental time window required for *SYCP1* activation, further substantiating that the deactivation of these locus-specific upstream regulators is one of the primary causes for the failed activation of the *SYCP1* gene. Notably, a concurrent scRNA-seq study has explicitly reported the downregulation of *SYCP1* expression in NOA patients (Chen et al., 2023).

### The dynamics of chromatin accessibility at meiotic DNA DSB hotspots are affected in some NOA patients

When investigating the differences in gene activity between NOA patients and normal Z stage spermatocytes, we observed that certain genes associated with meiotic DNA DSB formation and repair (Bhattacharyya et al., 2019), such as *SPO11*, *DMC1*, and *RAD51*, were downregulated in some patients (NOA5, NOA6, NOA13, and NOA14 patients) (Fig. 6A). Notably, approximately one-quarter of the cells exhibited nearly zero activity levels for these genes. Additionally, within the same individual patient, cells with concurrent downregulation of all three genes constituted only a small fraction, while a larger number of cells showed downregulation in any one of these three genes (Fig. S8A).

**Figure 6.**
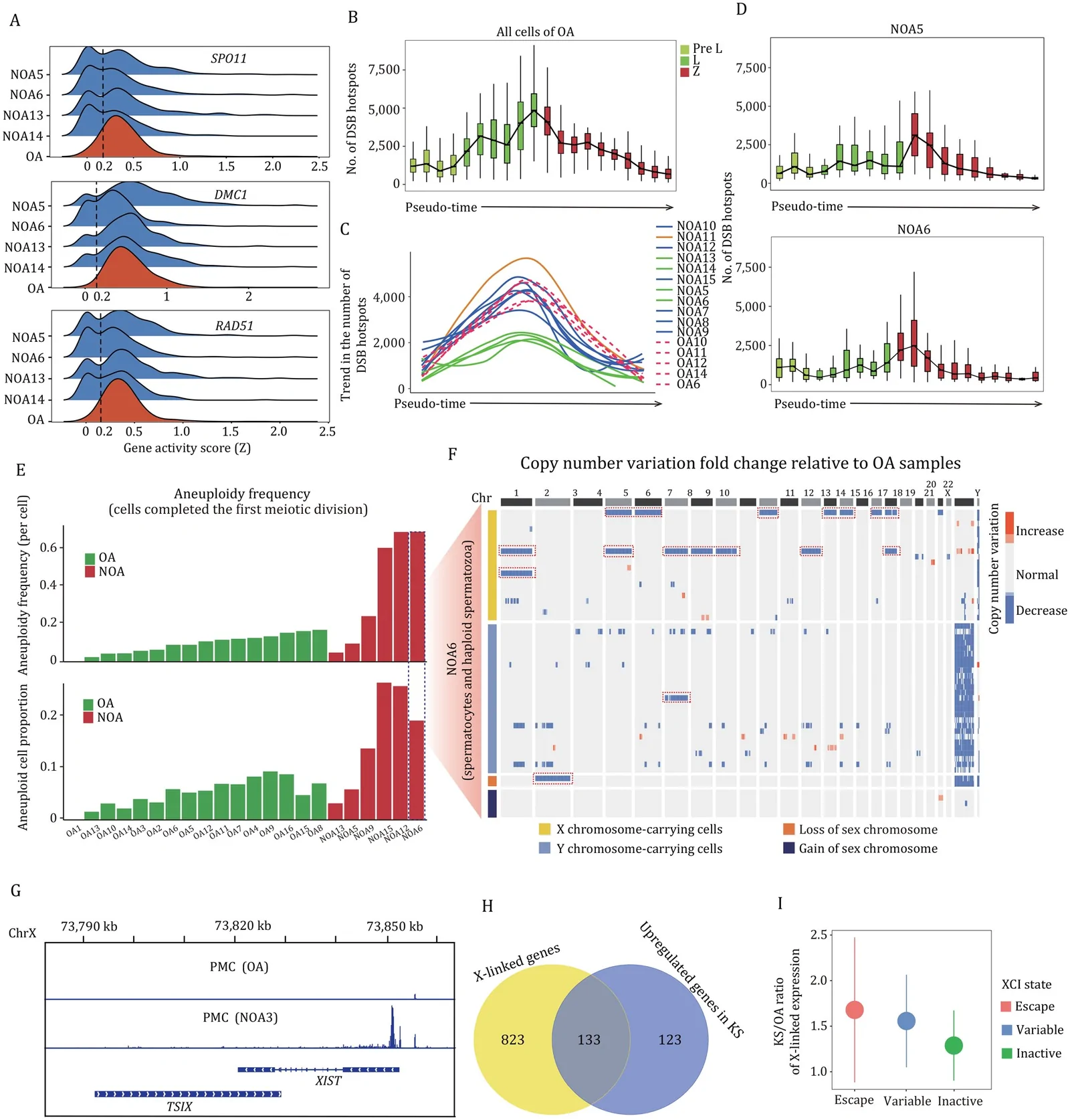
**Disruption of chromatin accessibility patterns at meiotic DNA DSB hotspots in NOA patients**. (A) Representative gene activity scores at the Z stage in OA (controls) and NOA5/6/13/14 patients. (B) Number of meiotic DNA DSB hotspots covered by each cell from OA samples along the trajectory from the PreL to Z stages. (C) Number of meiotic DNA DSB hotspots covered by each cell from OA and NOA patients along the trajectory from the PreL to Z stages, fitted with smoothed curves. (D) Number of meiotic DNA DSB hotspots covered by each cell from NOA5 and NOA6 patients. (E) Proportion of aneuploidy following the first meiotic division. The frequency of aneuploid events per cell (top) and the proportion of aneuploid cells (bottom) are shown. (F) Heatmap depicting copy number variations (CNVs) in NOA6 cells following the first meiotic division (only regions > 15 Mb are displayed). (G) IGV tracks displaying chromatin accessibility in PMCs near the *XIST* and *TSIX* genes in a KS patient (NOA3) compared to controls (OA). (H) Overlap between X-linked genes and upregulated genes in the KS patient (NOA3). (I) Relative activity scores of three types of X-linked genes in the KS patient (NOA3) compared to controls (OA) in PMCs.

Next, we employed the same method as mentioned above to calculate the number of peaks overlapping with meiotic DSB-hotspot regions in each germ cell. We sorted and categorized the cells into 20 groups based on a pseudotemporal trajectory and counted the number of DSB-hotspots in each group (Fig. 6B). Interestingly, NOA5, NOA6, NOA13, and NOA14 exhibited dynamic changes in DSB patterns that differed significantly from those of other NOA patients and OA controls (Fig. 6C). These four patients showed no discernible peak in the distribution of DSB-hotspot numbers, with similar median values across these 20 groups. For instance, in NOA6 and NOA5 patients, the highest median number of DSB-hotspots with open chromatin features across these 20 germ cell groups was only around 2,000 and 3,000, respectively, which is only half of that observed in the control group (OA) (Fig. 6D). This suggests that in some spermatocytes of these four patients (NOA5, NOA6, NOA13, and NOA14), the number of meiotic DSB-hotspot regions with open chromatin features strongly decreased. Previous research has reported a reduction in the number of crossovers in male infertility patients, suggesting a potential association between the reduced chromatin accessibility at these hotspots and the defects in genuine DSB formation (Ferguson et al., 2007).

### Increased aneuploidy of sperm cells in NOA patients

Previous studies have indicated that NOA patients have several times more aneuploid sperm cells than normal individuals (Ferguson et al., 2007; Vozdova et al., 2012). In our investigation, NOA patients have been found to exhibit defective chromatin accessibility at meiotic DNA DSB hotspots and impaired synaptonemal complex formation, which may disrupt the accurate segregation of chromosomes during meiosis, resulting in aneuploid sperm. Among the 12 NOA patients with germ cells in our collected samples, 6 patients were detected with over 30 germ cells completing the first meiotic division. Our findings revealed a notable increase in both the proportion of aneuploid cells and the frequency of aneuploid events per cell in NOA6, NOA12, and NOA15 compared to OA, approximately 2 to 3 times higher (Fig. 6E). For example, in the NOA6 sample, we identified 52 germ cells completing the first meiotic division, among which 18 autosomal loss events and 1 autosomal gain events from 6 sperm cells were identified; 2 sex chromosome loss events and 5 sex chromosome gain events from 7 sperm cells were identified (Figs. 6F and S8B). Our results demonstrate that the chromatin accessibility landscape in NOA patients is predominantly arrested at the zygotene stage. Even when a rare fraction of germ cells manages to complete the first or second meiotic division, these unresolved epigenetic anomalies remain strongly associated with chromosome segregation errors, contributing to a significantly elevated proportion of aneuploid spermatozoa.

### Chromatin accessibility of PMC in the testes of NOA patients

Peritubular myoid cell (PMC), key components of the seminiferous tubule wall, exhibit smooth muscle-like characteristics and play a crucial role in intra-testicular sperm transport (Zhou et al., 2019). Evidence suggests their close involvement in spermatogenesis. The structure and function of PMC can be changed when spermatogenesis is abnormal (Mayerhofer, 2013). The tubular wall structure often changes in men with impaired spermatogenesis, accompanied by alterations in extracellular matrix (ECM) deposition and peritubular cell morphology, suggesting fundamental changes in peritubular cell function (Volkmann et al., 2011).

Although testicular fibrosis is not a universal feature of all azoospermic patients, in our collected NOA cohort, PMC from Class II, Orchitis, SCOS, and KS patients exhibited significant differences in chromatin accessibility features compared to OA controls (FDR < 0.05 and abs(Log_2_FC) ≥ 0.5), identifying 2699, 3516, 91, and 183 aberrantly opened DAPs; and 2434, 2570, 1280, and 2350 aberrantly closed DAPs, respectively (Fig. 5A). GO enrichment analysis on these DAPs revealed varying degrees of enrichment in terms such as “Hyaluronan catabolic process” and “Positive regulation of smoothened signaling pathway” for aberrantly closed DAPs (Fig. S9B). Aberrantly opened DAPs were enriched in terms like “Collagen-activated signaling pathway” and “Collagen-activated tyrosine kinase receptor signaling pathway” (Fig. S9B). Moreover, the gene activity score of associated genes was disrupted (Fig. S9A), indicating altered chromatin accessibility features in the PMC of some NOA patients.

KS is caused by a 47, XXY karyotype. Studies have shown that the extra X chromosome in the somatic cells of KS patients becomes inactivated due to elevated expression of *XIST* (Mahyari et al., 2021). In our research, we observed that the *XIST* promoter region in PMC of KS patients exhibited significantly increased chromatin accessibility compared to controls, indicating *XIST* expression and suggesting that the extra copy of X chromosome may be inactivated in PMC (Fig. 6G). While we observed that most X-linked genes were indeed silenced, some showed increased gene activation scores compared to the control group, suggesting the presence of escape from inactivation (Fig. 6H). Based on the status of X-chromosome inactivation (XCI) in somatic cells, X-linked genes can be classified into four categories: inactive, escape, variable, and unknown (Lu et al., 2024; Tukiainen et al., 2017). We compared the proportion of gene activity scores among these three types of X-linked genes between OA and KS patients (Fig. 6I). The results showed approximately a 68% increase in the activity scores of escape X-linked genes, a 55% increase in variable X-linked genes, and 29% increase in inactive X-linked genes, indicating that the whole X chromosome is only partially inactivated and the escaped X-linked genes indeed escape from inactivation of the extra X chromosome in the PMC of KS patients.

### scATAC-seq improves the interpretation of genetic risk variants for male infertility

Previous GWAS on male infertility have identified numerous genetic risk signals. Among these, the MHC class II region has been consistently reported as the most strongly associated genomic locus for NOA across Chinese, Japanese, and European populations (Cerván-Martín et al., 2022; Hu et al., 2011, 2014; Kanai et al., 2018). To integrate these genetic findings with our epigenetic landscape, we curated a compendium of 12 previously reported lead SNPs within the MHC II region and expanded them to 538 variant candidates based on linkage disequilibrium (LDproxy, *R*^2^ > 0.7) (Table S5; Fig. S10A). Furthermore, our comparative analysis of Z stage spermatocytes from three NOA subtypes versus OA controls revealed a marked concentration of DAPs within this MHC II region, specifically surrounding the HLA-DRB5 and HLA-DRB1 genes (Fig. 7A). Notably, this locus was also characterized by extensive long-range interactions among its candidate *cis*-regulatory elements (cCRE) (Fig. 7A).

**Figure 7.**
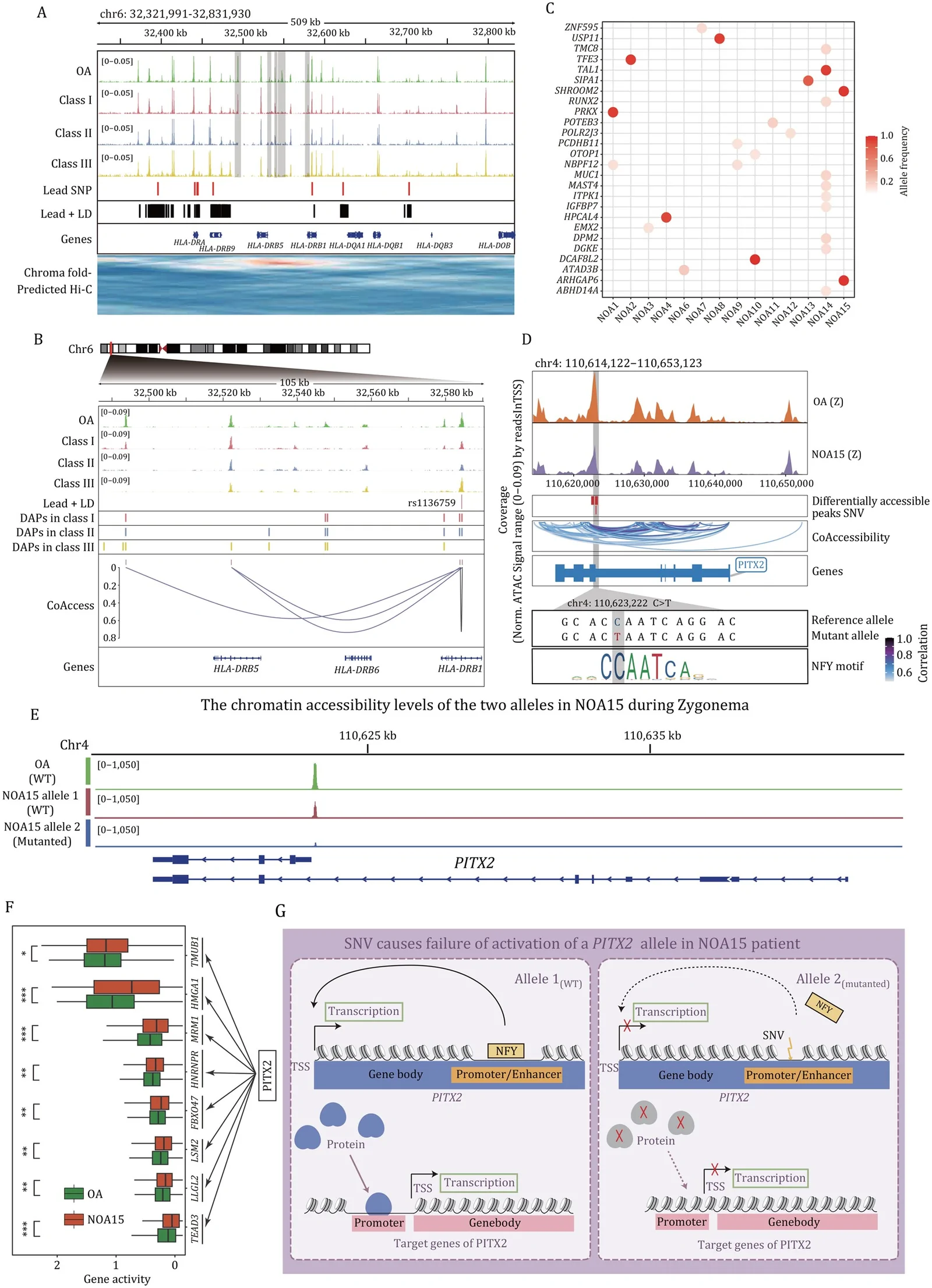
**Genomic variations (GWAS loci and SNVs) in NOA patients**. (A) DAPs between each NOA class and OA within the chr6:32,321,991–32,831,930 region, including lead GWAS loci and their LD-expanded loci, along with the associated genes and the Hi-C structure of this region predicted using ChromaFold. (B) The GWAS SNP rs1136759 overlaps with DAPs, and the overlapping DAPs exhibit strong co-accessibility with other DAPs. (C) SIFT-predicted functionally deleterious SNV site. (D) In patient NOA15, a heterozygous C-to-T mutation within the proximal promoter/enhancer (*cis*-regulatory element) of the *PITX2* locus disrupts the binding motif of the NFY activator complex, leading to localized chromatin closure around this SNV site during the Z stage. (E) IGV tracks showing peaks of mutant allele and wild-type allele in the *PITX2 cis*-regulatory element region. (F) In patient NOA15, the activation levels of eight potential target genes of PITX2 were significantly downregulated (Wilcoxon test, *P < 0.05, **P < 0.01, ***P < 0.001). (G) Schematic of the SNV-induced dysregulation of *PITX2*. An SNV within the proximal promoter/enhancer (*cis*-regulatory element) of the *PITX2* locus disrupts the motif of the NFY activator complex. This disruption leads to localized chromatin closure and the failure of epigenetic activation for the mutant *PITX2* allele. Consequently, this causes a global reduction in downstream PITX2 motif activity, thereby decreasing the inferred gene activity of its meiotic target genes.

Interestingly, we identified a specific cCRE overlapping with the GWAS risk SNP rs1136759, which exhibited significantly reduced chromatin accessibility during the Z stage in both Class I and Class II NOA patients. In normal OA controls, this cCRE maintains a highly open chromatin state during the Z stage and exhibits significant long-range interactions with other cCREs (Fig. 7B). These findings suggest that such GWAS risk variants may disrupt the accessibility and regulatory networks of critical cCREs in Z-stage spermatocytes, potentially contributing to the spermatogenic arrest observed in these patients.

### Point mutations in cis-regulatory elements of the genomes of NOA patients

Because ATAC-seq specifically captures reads from open regulatory regions, it serves as a powerful and cost-effective strategy for profiling functionally critical non-coding sequences (Massarat et al., 2021). Calling single-nucleotide variants (SNVs) directly from these active regulatory landscapes provides a dual advantage. First, it enables the discovery of regulatory SNVs that disrupt TF binding motifs and enhancer functions. Second, it facilitates the detection of non-synonymous mutations that alter protein-coding sequences within actively accessible regions (Fig. S10B). Using OA patients as baseline controls, we identified highly confident SNVs (supported by more than 3 cells) across all cell types in our 15 NOA patients, with the mutational burden ranging from 9 (in NOA4) to 292 (in NOA14) SNVs (Fig. S10C). Notably, these variants were predicted to exert severe deleterious functional effects on 26 distinct genes (Ng and Henikoff, 2003) (Fig. 7C). Furthermore, by assessing the impact of these SNVs on TF binding motifs using FABIAN-variant (Steinhaus et al., 2022), we discovered that 11 NOA patients harbored mutations leading to significantly diminished binding activities of 176 TFs (Fig. S10D). Crucially, this included master regulators intimately involved in meiosis, such as CTCF, OVOL1, USF1, NFYA, and NFYC.

### Point mutations as the genetic basis for the dysregulation of PITX2 in the NOA15 patient

In patient NOA15, we observed a significant decrease in the inferred gene activity of *PITX2* during the Z stage (Fig. S10E). As a crucial transcription factor for human primary spermatocyte development (Fig. S10F), the PITX2 gene encodes multiple isoforms with distinct biological functions. Notably, recent rodent studies have demonstrated that a specific *PITX2*transcript is significantly upregulated during meiotic prophase to support active meiosis, and its suppression is intrinsically linked to the inhibition of testis development (Nandi et al., 2011; Tian et al., 2023).

To uncover the epigenetic etiology of this deficiency, we identified a heterozygous SNV (chr4:110623222, C > T) within a strong ATAC-seq peak at the *PITX2* locus. According to the Ensembl regulatory build and ENCODE annotations (Abascal et al., 2020; Zerbino et al., 2015), this genomic region functions as a crucial proximal cCRE with validated promoter/enhancer activity specifically in human testis tissue. Our motif analysis predicted that this SNV disrupts the binding motif of the NFY activator complex within this highly conserved element (Fig. 7D).

To further investigate the impact of this heterozygous point mutation, we conducted an allele-specific genotyping analysis on the scATAC-seq fragments within this cCRE. We found that the number of fragments containing the mutated allele was significantly lower—approximately one-quarter—than that of the wild-type allele (Fig. 7E). This demonstrates that the disruption of the NFY motif causes localized chromatin closure, subsequently depriving the mutant *PITX2* allele of essential regulatory activation during the zygotene stage.

Consequently, this upstream epigenetic silencing led to the systemic failure of the *PITX2*-driven meiotic network. Meanwhile, we observed a specific and global reduction in the activity of PITX2 motifs in NOA15 spermatocytes during the Z stage (Fig. S10E), strongly inferring a depletion of functional PITX2 protein. Based on our established regulatory network for normal sperm development, we predicted a critical downstream target gene module of PITX2, including *TMUB1*, *HMGA1*, *MRM1*, *HNRNPR*, *FBXO47*, *LSM2*, *LLGL2*, and *TEAD3*. In patient NOA15, these target genes exhibited significantly reduced gene activity scores at the Z stage (Fig. 7F). Taken together, the heterozygous mutation compromises *PITX2* activity by abolishing NFY complex binding at its proximal promoter/enhancer, which in turn precipitates the comprehensive silencing of its downstream meiotic target network, and likely represents one of the key contributing factors to the spermatogenic abnormalities observed in this patient (Fig. 7G).

## Discussion

Spermatogenesis is intricately linked to epigenetic regulation. Although previous studies utilized standard 10x Genomics scATAC-seq platforms to explore chromatin accessibility during human spermatogenesis, the limited fragment capture rate often obscured fine-scale regulatory dynamics. In this study, we employed an ultra-high-quality scATAC-seq technique to overcome this limitation, capturing over 70,000 fragments per single cell. This superior resolution enabled us to identify over 810,000 candidate cis-regulatory DNA elements using a relatively modest number of single cells, maximizing the retention of open chromatin information. The most profound manifestation of this technological advantage lies in our characterization of meiotic homologous recombination. The formation and repair of DNA DSBs are highly transient events. Sparse epigenomic data typically fail to capture these intermediate states. However, our exceptional capture rate allowed us, for the first time, to trace the complete epigenetic lifecycle of meiotic DSB hotspots—specifically, the dynamic opening and closing of chromatin accessibility—at single-cell resolution. We revealed a broad yet strictly regulated epigenetic window: during this period, a single normal spermatocyte establishes an open chromatin state at thousands of DSB hotspots, although ultimately only a small fraction proceeds to form physical breaks and crossovers.

Building upon this high-resolution baseline, our study uncovered the epigenomic defects and the systemic collapse of epigenetic regulation in iNOA—a phenomenon we characterize as temporal dysregulation and regulatory decoupling. Spermatogenesis requires master TFs to operate in a highly synchronized, sequential sequence; they must be precisely activated to drive cellular differentiation and promptly repressed to prevent interference with subsequent developmental stages. Our analysis reveals that in NOA patients, crucial TFs (such as FOXD3) fail to function at the correct developmental stages. We speculate that this upstream regulatory failure prevents the essential chromatin opening at the cis-regulatory regions of downstream target genes required for meiosis. For instance, this epigenetic dysregulation is strongly associated with the activation failure of key meiosis-related genes like *SYCP1* and *BUB1*. Ultimately, the collapse of this TF regulatory network disrupts synaptonemal complex assembly, arresting spermatogenesis predominantly at the zygotene stage. Even if a rare fraction of germ cells bypass this zygotene arrest to form haploid spermatozoa, they carry a significantly higher burden of aneuploidy. This highlights a critical, previously underappreciated genetic risk: the utilization of surgically retrieved spermatozoa from NOA patients for *in vitro* fertilization may harbor substantial genetic liabilities.

Furthermore, our study elucidates the profound clinical value of exploring the non-coding genome. Current diagnostic paradigms for male infertility rely heavily on microscopic morphological evaluations or whole-exome sequencing, the latter of which systematically ignores 98% of the genome. By directly mapping the cCRE landscape, our scATAC-seq approach serves as a functional filter, successfully identifying pathogenic SNVs directly within active regulatory elements. We demonstrated that a single point mutation in the cis-regulatory region of a master TF, such as *PITX2*, is sufficient to disrupt upstream TF binding and trigger a devastating downstream cascade, ultimately leading to spermatogenic arrest. This establishes a new paradigm for molecular diagnostics: profiling chromatin accessibility allows us to assign definitive functional significance to non-coding variants that would otherwise be classified as variants of unknown significance.

We acknowledge that scATAC-seq has inherent limitations; for example, homozygous mutations that completely abolish chromatin accessibility may render the affected regulatory regions undetectable in this assay. In such cases, integrating scATAC-seq with whole-genome sequencing remains indispensable for comprehensive mutational profiling. Nevertheless, by achieving unprecedented data quality, this study provides a robust, high-resolution theoretical framework that shifts the focus of male infertility research from descriptive phenotypes to precise, network-level epigenetic mechanisms. This deeper understanding paves the way for advanced genetic screening and the development of targeted therapeutic interventions for non-obstructive azoospermia.

## Supplementary Material

pwag033_Supplementary_Data

## Data Availability

All sequencing data of scATAC-seq generated in this study have been deposited in the Genome Sequence Archive for Human (GSA Human, ngdc.cncb.ac.cn/gsa-human/) under accession number HRA007344.

## Code availability

The analysis code is deposited in GitHub (github.com/hlxie/Human_Spermatogenesis).

## Abbreviations

AZF: Y chromosome azoospermia factor
cCRE: candidate cis-regulatory element
D: diplotene
DAP: differential accessibility peak
Diff.ing SPG: differentiating spermatogonia
DSB: DNA double-strand break
ECM: extracellular matrix
FC: fold change
FDR: false discovery rate
GRN: gene regulatory network
GWAS: genome-wide association study
iNOA: idiopathic non-obstructive azoospermia
KS: Klinefelter syndrome
L: leptotene
MHC: major histocompatibility complex
MSCI: meiotic sex chromosome inactivation
NOA: non-obstructive azoospermia
OA: obstructive azoospermia
P: pachytene
PMC: peritubular myoid cell
PreL: preleptotene
PreP: pre-pachytene
S (S1, S2, S3): spermatid (spermatid subpopulations)
scATAC-seq: single-cell assay for transposase-accessible chromatin with sequencing
SCOS: Sertoli cell-only syndrome
scRNA-seq: single-cell RNA sequencing
SNV: single nucleotide variant
SPC7: secondary spermatocyte
TF: transcription factor
TSS: transcription start site
Undiff.SPG: undifferentiated spermatogonia
XCI: X-chromosome inactivation
Z: zygotene
