# Supplementary material for: Epigenetic dynamics of human spermatogenesis and their dysregulation in non-obstructive azoospermia
Source: Protein Cell. 2026 May 22;17(10):934–53. doi: 10.1093/procel/pwag033 (PMC13626738; doi:10.1093/procel/pwag033)
Supplement: Supplementary file 1 [file pwag033_supplementary_data.zip › Supplementary_Data/Supplemental materials.pdf]

## Materials and methods

**Single cell suspension preparation**

Testicular biopsy retrieved from patients with azoospermia by testicular sperm aspiration (TESA) (OA) or microdissection testicular sperm extraction (mTESE) (NOA). The biopsied testicular tissues from TESA procedures were washed three times with Dulbecco's Modified Eagle Medium (DMEM, C11995500BT, Thermo Scientific) containing 10% fetal bovine serum (FBS, 10437028, Gibco) and then minced with sterile scissors. Subsequently, 500 μL of Accutase cell dissociation solution (A6964, Sigma-Aldrich) was added, and the samples were incubated at 37°C for 15 minutes. To ensure complete tissue digestion into single cells, the mixture was pipetted 30 times at 7 minutes and at the end of digestion. The cell suspension was filtered through a 40 μm pre-separation filter, centrifuged at 300×g for 8 minutes, and the cells were resuspended in 500 μL of DMEM (containing 10% FBS).

**Library construction for scATAC-seq analysis**

In each reaction, the collected cells underwent a wash with ice-cold PBS to prevent potential contamination. Subsequently, the cells were pelleted by centrifugation at 500 × g for 5 minutes at 4 °C, and the supernatant was carefully removed. The cell pellets were then resuspended in Omni-ATAC Lysis Buffer—composed of 10 mM Tris-HCl (pH 7.4), 10 mM NaCl, 3 mM $MgCl_2$ , 0.1% IGEPAL CA-630, 0.1% Tween-20, and 0.01% digitonin—and incubated on ice for 5 minutes. Following lysis, the nuclei were immediately centrifuged at 800 × g for 5 minutes at 4 °C. The resulting nuclear pellets were resuspended in 50 μL of Tagmentation Mix, which included 33 mM Tris-acetate (pH 7.8), 66 mM potassium acetate, 10 mM magnesium acetate, 16% dimethylformamide, 0.01% digitonin, and 10 μL of 8.3 μM Tn5 transposase. In this study, the Tn5 transposase (sourced from Novoprotein) contained a universal adaptor sequence: TCGTCGGCAGCGTCAGATGTGTATAAGAGACAG.The reaction was incubated on thermomixer at 37 °C, 800 rpm, for 30 minutes and stopped by adding 50 μl Tagmentation Stop Buffer (10 mM Tris-HCl, pH 8.0, 20 mM EDTA). Nuclei were stained with DAPI after tagmentation, and single nucleus was sorted by MoFlo XDP Cell Sorter (Beckman Coulter) into each well of the 96-well plate containing 4 μl Lysis Buffer (50 mM Tris-HCl, pH 8.0, 50 mM NaCl, 20 μg/mL Proteinase K, 0.2% SDS, 10 μM S5xx/N7xx Nextera Index Primer Mix, 5 μM each). Incubate the plate at 65 °C for 15 minutes to release the Tn5 transposase and disassociate

nucleosomes from chromatin. Add 4μl 10% TWEEN-20 to each well to quench SDS. 20μl PCR mixture was prepared by adding 10μl 2X KAPA HiFi HotStart ReadyMix (Kapa Biosystems) and 2μl H2O to each well. Perform the PCR procedure as follows: 72 °C 10 min; 98 °C 5 min; [98 °C 10s, 63 °C 30s, 72 °C 20s] x 20 cycles; 4°C hold. The amplified libraries from each well of the 96-well plate were pooled into a single tube, then purified and concentrated using the Qiagen MinElute PCR Purification Kit. Fragment size selection was performed with SPRIselect beads (Beckman Coulter), first applying a 0.5× volume for the upper cutoff, followed by a 1.2× volume for the lower cutoff. The libraries were then combined and subjected to 150 bp paired-end sequencing on the Illumina NovaSeq 6000 platform at Novogene Co., Ltd.

**In situ karyotyping**

In situ karyotyping of human testicular tissues was performed using a modified version of our previously established method. Briefly, paraffin-embedded sections of human testes (8 μm thick) were incubated overnight at 4 °C with rabbit anti-SYCP3 antibody (rabbit, 1:300, ab15093, Abcam) and mouse anti-γH2AX antibody (mouse, 1:200, ab26350, Abcam). SYCP3 labeling marked the synaptonemal complex to distinguish meiotic stages of germ cells, whereas the staining pattern of γH2AX indicated DNA damage and repair status to infer meiotic progression. The sections were subsequently counterstained with HOE dye (1:100 dilution) for nuclear localization and identification. After staining, the sections were sealed by an anti-fade mounting buffer and were acquired by an Andor Dragonfly spinning-disc confocal microscope equipped with a scientific complementary metal-oxide semiconductor (sCMOS) camera (Andor Zyla 4.2), a × 100, 1.44 N.A. oil objective, and the 405-nm (HOE) , 488-nm (anti-γH2AX) and 561-nm (anti-SYCP3) lines of the Andor Integrated Laser Engine (ILE) system with a spinning-disc confocal scan head (Andor Dragonfly 500). The sections were imaged with laser 405-nm, 488-nm and laser 561-nm around 10–15%, exposure time 100–200 ms. Images were acquired by the Fusion 2.1 software (https://andor.oxinst.com/products/dragonfly#fusion).

After the acquisition, images were processed by ImageJ (http://rsbweb.nih.gov/ij/) for the projection of all z-stacks and merged color channels, and the 3D images were used to identify the meiotic stages of germ cells. The detail indexes of identification are listed here: leptotene, abundant chromatin fibrils appear in nucleus; zygotene,

chromosome, and classical tripartite synaptonemal complex structure are clearly visible; and pachytene, the chromosomes are the shortest and thickest. Homologous chromosome association and homologous recombination are completed; diplotene, the homologous chromosomes begin to separate, decondensed and diffuse from each other; and 2 to 4 bright spots appear in the nucleus. To clearly highlight the meiotic stage of germ cells, the γH2AX (488 nm) and SYCP3 (561 nm) channel was inverted to black and white (B/W) by the ImageJ software in Fig. S6.

**Quality control, dimensionality reduction and clustering of scATAC-seq data**

Raw sequencing data were trimmed for adaptors using cutadapt (v3.2) and then aligned to the hg38 reference genome using bowtie2 (v2.2.1), and low quality (MAPQ < 30) alignments were further removed with samtools (v1.11). The bam files were converted to fragment files of each cell were generated using sinto (v0.7.2.2, https://github.com/timoast/sinto) with default parameters. We followed the ArchR (v1.0.2) pipeline to perform predominantly downstream analysis of scATAC-seq data. Quality control metrics were computed for each single cell. Only cells with TSS enrichment score >= 4 and number of unique fragments >= 10,000 were retained. After peak calling (using MACS2 (v 2.2.7.1)), a cell-by-peak matrix was computed. We then used ArchR's implementation of iterative LSI dimensionality reduction using the 'addIterativeLSI' function. Finally, UMAP embedding was then computed in the first 30 dimensions of LSI from the peak matrix, and graph-based clustering algorithm were used to identified cell clusters. We identified marker genes for each cluster using the 'getMarkerFeatures' function with the accessibility around each gene (the 'Gene Activity Score') as a proxy for gene expression. The cell-by-peak matrix was binarized and used for identifying differential peaks for each cell cluster using 'getMarkers' function.

**Identification of X- and Y-bearing haploid cells**

To accurately distinguish between X-bearing and Y-bearing germ cells following the first meiotic division (encompassing both secondary spermatocytes and haploid spermatids), we evaluated the relative abundance of chromatin accessibility signals on the sex chromosomes. For each post-meiotic cell, we calculated the ratio of normalized total scATAC-seq reads mapping to chromosome Y over those mapping to chromosome X (chrY/chrX read ratio). Because homologous sex chromosomes

segregate during the first meiotic division, this metric effectively separates the two distinct populations. Based on the bimodal distribution of these ratios across all secondary spermatocytes and spermatids, we established stringent empirical thresholds to assign cell identities with high confidence. Specifically, cells exhibiting a chrY/chrX read ratio strictly greater than 0.15 were classified as Y-bearing cells, whereas cells with a ratio of less than 0.05 were classified as X-bearing cells. Cells with intermediate ratios falling between these two strict thresholds were considered ambiguous.

**ChromVAR motif analysis**

The TF accessibility profiles across single cells were computed using chromVar (v 1.16.0), which generated a cell-by-motif matrix with GC bias-corrected chromVar deviation scores were utilized as metrics for global TF motif activity. Catalog of Inferred Sequence Binding Preferences (CIS-BP) motifs from chromVar motifs 'human_pwms_v1' were matched to scATAC peaks using the matchMotif function of the motifmatchr package.

**Trajectory analysis for human spermatogenesis**

Given the known developmental sequence of spermatogenesis, we employed a semi-supervised approach to delineate the trajectory of spermatogenesis at the single-cell level. Using the 'addTrajectory' function from ArchR with the trajectory backbone parameter 'Undiff.SPG-1, Undiff.SPG-2, Diff.ing SPG-1, Diff.ing SPG-2, PreL, L, Z, PreP, P, D, SPC7, S1, S2, S3' to create the trajectory. ArchR aligns all cells to the trajectory based on their Euclidean distance to the nearest point along the manifold and then scales this alignment to 100, assigning a numerical label between 0 and 100 to each cell for sorting purposes. In subsequent analyses, we can examine changes in TF motif activity, gene activity scores, or other cell features across pseudotime to identify dynamic regulatory factors or elements throughout the entire cell trajectory.

**Integration of scRNA-seq and scATAC-seq datasets**

The cell-by-peak matrix from the ArchR Project was extracted as the input for Signac to create a Seurat Object, and then Gene activities were calculated using the Signac function 'GeneActivity' with the default parameters. For the scRNA-seq data, we

selected Smart-seq2 sequencing data obtained from samples collected in the same hospital and department. Specifically, we used the TPM matrix from the NCBI Gene Expression Omnibus (GEO) dataset GSE106487, which contains 2,854 cells representing 15 germ cell types and 3 somatic cell types. We used the Seurat R package to perform dimensionality reduction and clustering on this data, creating a Seurat Object. We selected 3000 variable genes identified in the scRNA-seq data and TFs with high variable motif activity identified in the scATAC-seq data as features to find integration anchors using the function 'FindIntegrationAnchors'. Then, we integrated the data using the 'IntegrateData' function with the parameter 'dims = 2:30'. Finally, we visualized the integrated data using UMAP and clustered the cells into 17 groups using a graph-based clustering algorithm. We identified the same major cell types in each dataset and demonstrated the correspondence between the labels from the scATAC-seq dataset and the scRNA-seq dataset.

**The establishment of regulatory networks using Pando**

We used the integrated Seurat Object from scATAC-seq and scRNA-seq as input for Pando to infer regulatory networks. To constrain the set of peaks considered by Pando, we utilized a set of conserved regions provided by Pando (phastConsElements20Mammals.UCSC.hg38). Within these regions, we scanned for TF motifs based on the motif database included in Pando, compiled from JASPAR and CIS-BP motifs, using the ˋfind_motifsˊ function. Subsequently, leveraging motif matches, single-cell-level log-normalized transcript counts, and cluster-level peak accessibility, we inferred the GRN using the Pando function ˋinfer_grnˊ with parameters ˋpeak_to_gene_method = 'Signac', upstream = 100,000, and downstream = 100,000ˊ. Based on the model coefficients returned by Pando, we constructed TF networks using the ˋfind_modulesˊ function with parameters ˋP_thresh = 0.01, rsq_thresh = 0.1, nvar_thresh = 10, and min_genes_per_module = 5ˊ. Finally, the 'plot_network_graph' function of Pando was used to visualize the inferred global GRN.

For cell type-specific GRN inference, we first filtered the edges of the global GRN using the parameters 'padj < 0.5 abs(corr) > 0.05'. Pseudo-bulk replicates were then generated for each cluster using the 'addGroupCoverages' function of ArchR, and the 'addReproduciblePeakSet' function of ArchR was utilized to produce peak sets for each cell type, which were used to further filter the edges of the global GRN. We

identified differential TF motif activity for each cell type using the 'getMarkers' function with parameters 'FDR <= 0.01 and mean Diff >= 0.5'. The calculated cell type-specific TF motif activity was then used to further filter the nodes of the global GRN. After filtering edges and nodes, we generated cell type-specific regulatory networks. For the inference of individual TF-regulated target genes, we required a stricter correlation threshold with the parameter 'abs(corr) > 0.3'.

**cisTopic modelling**

We used the peak-cell count matrix as input for cisTopic. The number of topics was varied from 5 to 50 at intervals of 5. The model was run for 500 iterations with an α of 50 divided by the number of topics and a β of 0.1. The most stable model, consisting of 14 topics, was selected based on topic coherence and log-likelihood in the final iteration. The region-topic scores were normalized to sum to 1 for each nucleus, and binarized topic lists were generated by assigning each region to the topic with the highest score. Each topic was then used as input for HOMER to perform motif enrichment analysis.

**Enrichment analysis for genomic region set overlapping**

The CGI and repeat element were downloaded from the UCSC table browser. Promoter regions were defined as regions 1 kb upstream and 0.5 kb downstream of transcription start sites (TSSs). Gene annotations were loaded from UCSC TxDb gene annotation of the hg38 genome in R using GenomicFeatures package. The significance of overlapping between different genomic region sets was evaluated using R package LOLA (v 1.19.1) (https://bioconductor.org/packages/release/bioc/html/LOLA.html). Briefly, after universe filtering and counting overlaps between query sets and reference sets, Fisher's exact test was utilized to evaluate the significance of region overlapping.

**Identification of meiotic DSB hotspots**

We analyzed DMC1 ChIP-seq data download from the NCBI Gene Expression Omnibus (GEO): GSE59836, and defined these regions as ssDNA-defined hotspot regions. The signal intensity of chromatin accessibility around ssDNA-defined hotspots centre was calculated using 'getFootprints' function of ArchR. To further refine the DSB hotspot regions, we intersected the ssDNA-defined hotspot regions with the open chromatin regions during the L and Z stages (the chromatin

accessibility of each cell type were called by ArchR's 'addReproduciblePeakSet' function), excluding areas within 2,000 bp of the TSS and the Y chromosome regions, and we defined these regions as DSB hotspot regions.

**Identification of cell types in NOA Samples**

To ascertain the cell identities of germ cells derived from NOA patients, we utilized LSI dimensionality reduction on both OA and NOA cell populations, followed by UMAP embedding in the first 30 dimensions of LSI. Subsequently, we employed the 'addClusters' function of ArchR with parameters 'resolution = 6, maxClusters = 60' to cluster the cells into 53 subgroups. Within each subgroup, OA cells displayed nearly identical cell type labels. Cell type labels observed in NOA cells within these subgroups were deemed consistent with those of OA cells.

**Identification of aneuploidy of sperm cells**

We specifically chose cells that had undergone the first meiotic division to identify cell CNVs (aneuploid sperm cells). The genome was partitioned into 5-Mb windows with a step size of 5 Mb using the slidingWindows function of GenomicRanges. These windows were then filtered against the ENCODE hg38 blacklist using the setdiff function in R. The number of insertions in each window was counted for each cell, resulting in a cell-by-window binarized matrix. Subsequently, the insertion count per window was computed for each filtered window. As a control, we also calculated the number of insertion reads for each window in all OA cells and grouped them by cell type. The mean of insertion reads for each window within each group served as a control. For each NOA cell, we selected the appropriate control based on its cell type to determine the presence of CNVs.

In the context of CNVs arising from chromosomal segregation anomalies, their lengths typically encompass whole chromosomes or chromosome arms. As such, we established specific criteria to discern whether a chromosome displayed losses or gains: an average fold change across the entire chromosome or chromosome arm of $<0.1$ or $>3.5$ indicated alterations. Regarding the identification of sex chromosomes that failed to segregate correctly, we defined criteria where the average fold change for both chrX and chrY exceeded 0.6. Given the inherent limitations in resolution with scATAC-seq for CNV detection, our analysis focused solely on chromosome arms of chr1 to chr8 and chr11 for assessing losses or gains.

**Co-accessibility analysis**

To identify potential cis-regulatory interactions and link distal regulatory elements to their putative target genes, we calculated the co-accessibility of chromatin peaks across single cells. This analysis was performed using the Cicero algorithm, which is natively implemented within the ArchR pipeline via the 'addCoAccessibility' function. By computing the correlation of accessibility between pairs of peaks, we established the regulatory linkages within non-coding chromatin module networks, particularly around disease-risk GWAS loci.

**Identification and analysis of SNVs in NOA patients**

For each OA individual's single-cell raw sequencing data, adaptor trimming was conducted using cutadapt (v3.2) before alignment to the hg38 reference genome via bowtie2 (v2.2.1). Subsequently, alignments with low quality (MAPQ < 30) were excluded using samtools (v1.11). The bam files from each OA sample were merged, and duplicates were marked using ‘MarkDuplicates’ function of Picard. Merged bam files were utilized for SNV detection with Mutect2. Following this, GATK (v4.4) functions 'GenomicsDBImport' and 'CreateSomaticPanelOfNormals' were utilized to establish the normal panel.

For each single cell raw sequencing data from NOA, adaptor trimming was performed using cutadapt (v3.2) and then aligned to the hg38 reference genome using bowtie2 (v2.2.1), followed by the removal of low-quality alignments (MAPQ < 30) using samtools (v1.11). The bam files from each NOA sample were merged, and duplicates were marked using the 'MarkDuplicates' function of Picard. SNVs were detected from the merged bam files using Mutect2 with the control panel of normals file from OA samples. Finally, the 'FilterMutectCalls' function of GATK was applied to filter SNVs.

To identify more reliable SNV sites, we only consider SNVs with greater than 3 cell supports in a single individual. To achieve this, we processed the BAM file of each single cell using the same procedure and retained SNV sites with support from at least 3 cells using the 'bcftools isec' function. Subsequently, we further filtered the SNVs using the 'bcftools filter -i' function with parameters 'AF>=0.1 && FILTER ==

"PASS"'. To eliminate common high-frequency SNP sites in the population, we utilized two databases, gnomAD (v2.1.1) (http://www.gnomad-sg.org/) and dbSNP (https://www.ncbi.nlm.nih.gov/snp/), to filter out SNP sites with AF>0.01, providing an additional refinement for the SNV sites of our NOA patients. This process resulted in the final set of SNV sites for each NOA patient.

In the subsequent analysis, we annotated the identified SNVs using the 'convert2annovar.pl' and 'annotate_variation.pl' functions of ANNOVAR (https://annovar.openbioinformatics.org/en/latest/), selecting for non-synonymous mutation sites. Additionally, we employed the FABIAN-variant software to predict the effect of DNA variants on transcription factor binding.

**Genotyping analysis of fragments based on specific SNV presence**

We used a heterozygous mutation site on the *PITX2* gene to genotype the reads covering this site. The specific steps are as follows: the VCF file containing all SNV sites of NOA15 and the bam file were used as input for extractHAIRS, and the resulting output was passed to HAPCUT2 for phasing of the SNV sites. Finally, we used whatshap haplotag to genotype the bam file of NOA15 at the specific SNV site. The bam files containing mutant and wild-type reads were then extracted separately, and bedtools genomecov and bedGraphToBigWig were used to convert the bam files to bw files for visualization in IGV.

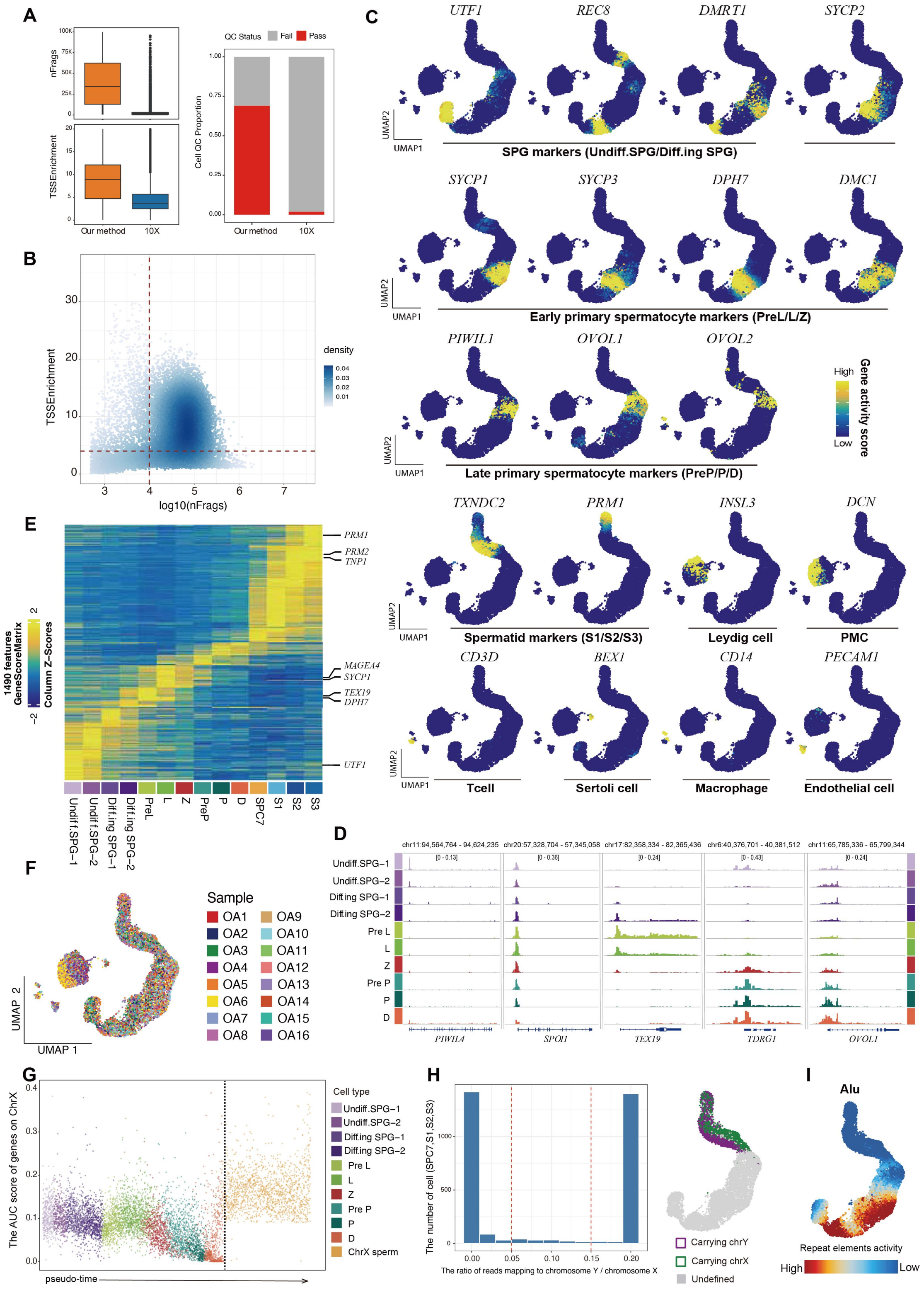


**Supplementary Fig.1 | Quality control of scATAC-seq data in OA patients.**

**(A)**. Comparison of scATAC-seq data quality between our method and a standard 10x Genomics platform. Left panels: Boxplots showing Unique Fragments per Cell (nFrags) and Signal-to-Noise Ratio (TSS Enrichment) comparing the two methods.

Right panel: Stacked bar chart showing the proportion of cells passing the stringent quality control thresholds defined in this study in both datasets. **(B)**. Quality control metrics for each single cell in OA samples. Horizontal and vertical lines represent our quality standards: TSS enrichment >= 4; unique fragment count >= 10,000. **(C)**. UMAP plots colored by gene activity score for the annotated cell type-defining gene. **(D)**. Sequencing tracks of scATAC–seq peaks. From left to right, *PIWIL4*, *SPO11*, *TEX19*, *TDRG1*, *OVOL1*. The tracks have been normalized to the total number of reads in TSS regions. **(E)**. Gene activity scores heatmap for the cell type specific genes. **(F)**. Uniform manifold approximation and projection (UMAP) dimensionality reduction after iterative LSI of scATAC–seq data from 16 OA samples. Each dot represents a single cell, colored by individual patient samples. **(G)**. The R package AUCell was used to calculate activation scores for X chromosome genes in cells that had completed the first meiotic division. The distribution of AUC scores is shown. **(H)**. The ratio of reads mapping to chromosome Y over chromosome X for individual cells (SPC7, S1, S2, S3). **(I)**. UMAP plots of germ cells colored by Alu repeat element activity.

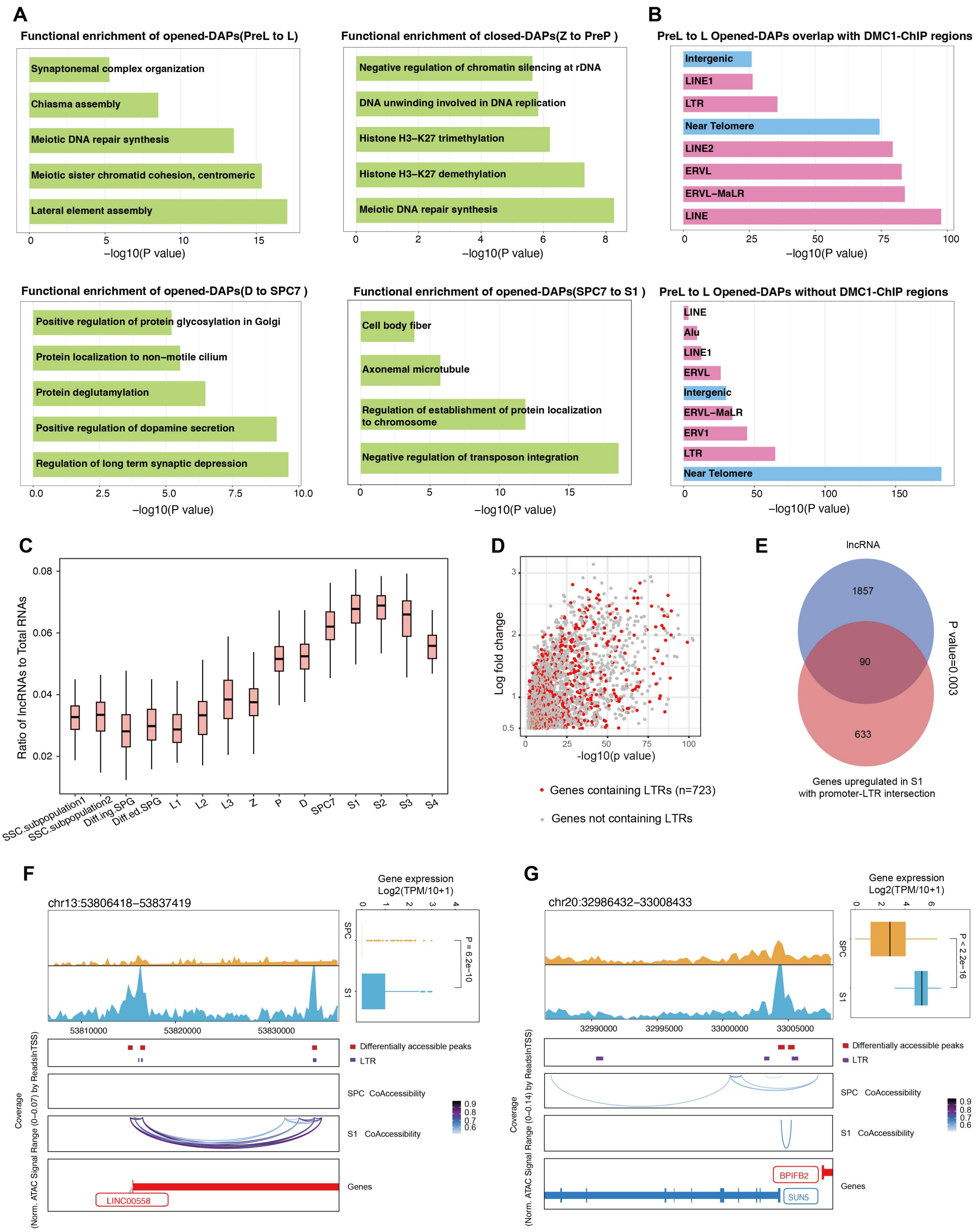


**Supplementary Fig.2 | Dynamics of chromatin accessibility profiles during human spermatogenesis.**

**(A)**. Functional enrichment for GO biological processes of the DAPs. **(B)**. Top: Enrichment analysis of genomic elements and repeat elements on PreL to L opened-DAPs overlap with DMC1-ChIP regions. Bottom: Enrichment analysis of genomic elements and repeat elements on PreL to L opened-DAPs without DMC1-ChIP regions.

**(C)**. The ratio of the number of expressed lncRNAs to the total number of expressed RNAs across the developmental trajectory. **(D)**. Genes upregulated in the S1 stage relative to the SPC7, red dots indicating the presence of LTRs in the gene's promoter region (n=723). **(E)**. Venn diagram showing that out of the 723 genes containing LTRs, 90 are lncRNAs (Fisher's exact test, p=0.003). **(F)**. Normalized pseudobulk tracks derived from scATAC-seq, DAPs between SPC7 and S1, locus of transposable element LTR and co-accessibility correlations in the *LINC00558* gene locus. **(G)**. Normalized pseudobulk tracks derived from scATAC-seq, DAPs between SPC7 and S1, locus of transposable element LTR and coaccessibility correlations in the *SUN5* gene locus.

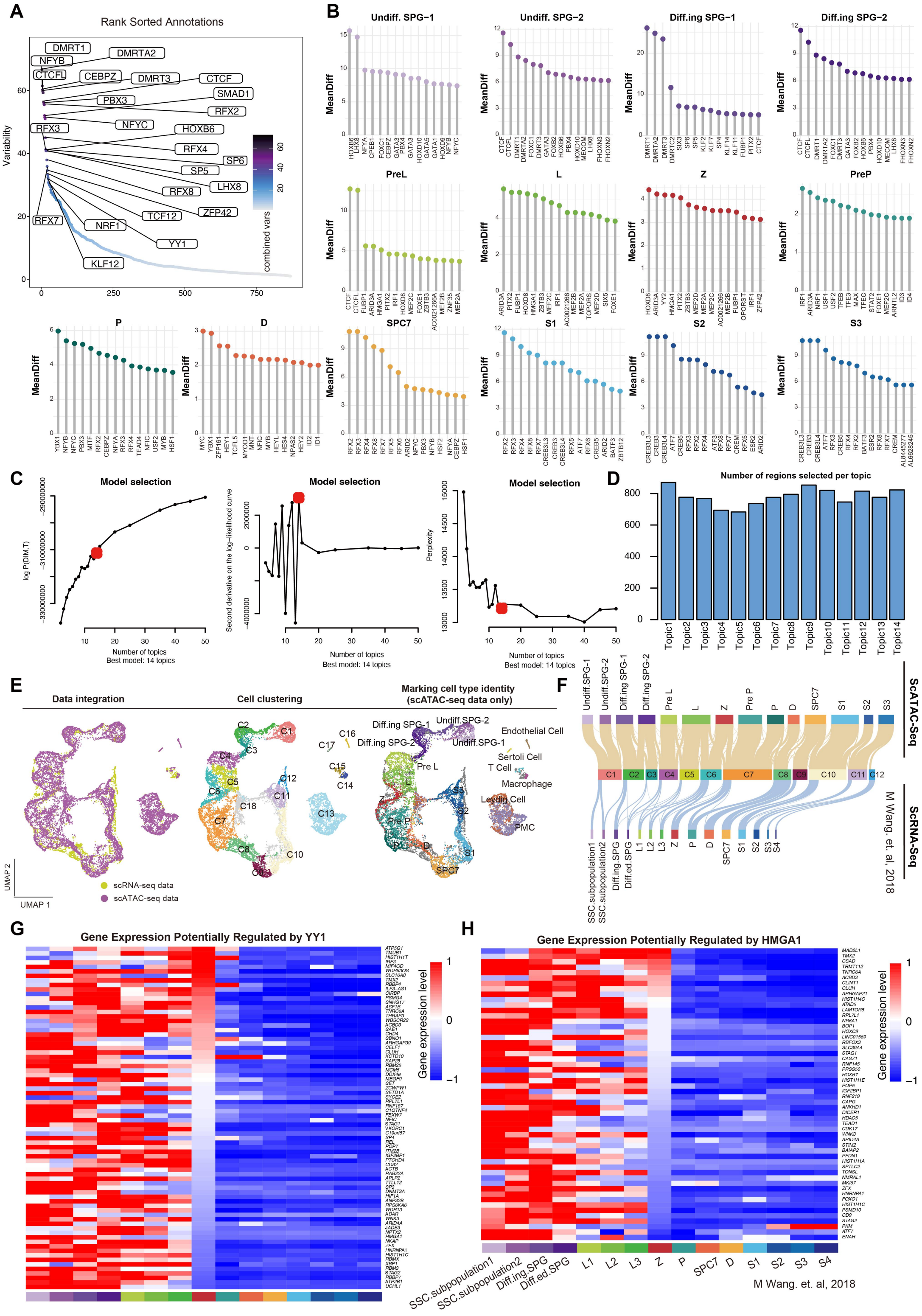

**Supplementary Fig.3 | The dynamics of TF motif activities across human spermatogenesis.**

**(A)**. TFs exhibiting the greatest variability in motif activity scores across

spermatogenesis. **(B)**. Top 15 cell type-specific activated TF motifs. **(C)**. Model selection and convergence curves from cisTopic. Models with 14 topics were selected as the optimal number of topics. **(D)**. Calculating the number of regions selected for each topic. **(E)**. Integration of scRNA-seq and scATAC-seq data. **(F)**. Correspondence between cell labels from scRNA-seq and scATAC-seq data. **(G)**. Gene expression pattern of YY1-regulated target genes during the Z stage. **(H)**. Gene expression pattern of HMGA1-regulated target genes during the Z stage.

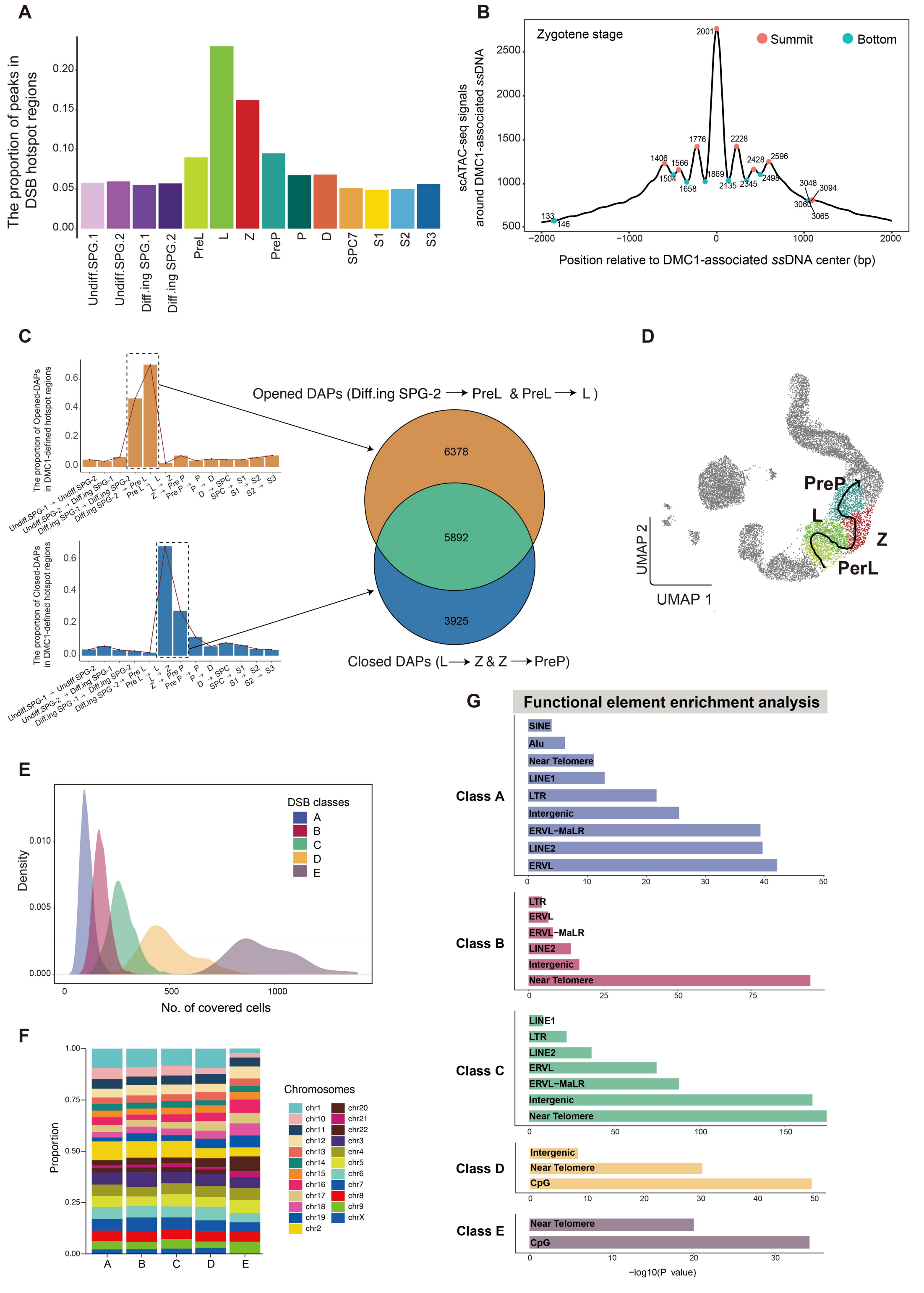


**Supplementary Fig.4 | Establishment of a binary matrix for meiotic DSB-hotspots at single-cell resolution.**

**(A)**. Bar plot shows the proportion of peaks overlapping with DMC1-defined hotspot regions for each cell type. **(B)**. ATAC signals around DMC1-defined hotspots centre

within ± 2 kb during the Z stage. Red dot indicates the highest point of the local region, blue dot indicates the lowest point, and numerical values indicate their positions relative to the DMC1-defined hotspots centre. **(C).** Overlap analysis between regions that newly gained accessibility (orange circle) and regions that lost accessibility (blue circle) across consecutive meiotic stages. The substantial intersection (green) robustly demonstrates the rapid opening and closing dynamics at these specific continuous DSB hotspots. **(D)**. Pseudotemporal trajectory from PreL to PreP in OA cells. **(E)**. Distribution densities of the five classes of DSB hotspots identified per cell. **(F)**. Five classes of DSB-hotspots distributions on each chromosome. **(G)**. Genomic feature enrichment analysis of five classes of DSB hotspots.

A

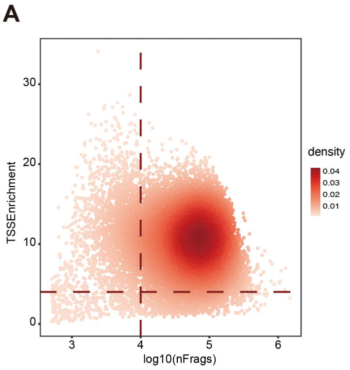


B

**The NO. of germ cells in NOA Patients**

| | NOA1 | NOA2 | NOA3 | NOA4 | NOA5 | NOA6 | NOA7 | NOA8 | NOA9 | NOA10 | NOA11 | NOA12 | NOA13 | NOA14 | NOA15 |
|---|---|---|---|---|---|---|---|---|---|---|---|---|---|---|---|
| Undiff.SPG-1 | 0 | 0 | 0 | 11 | 101 | 46 | 49 | 54 | 23 | 23 | 148 | 40 | 30 | 34 | 35 |
| Undiff.SPG-2 | 1 | 0 | 0 | 14 | 97 | 52 | 48 | 70 | 39 | 39 | 143 | 45 | 61 | 38 | 55 |
| Diff.ing SPG-1 | 0 | 0 | 0 | 11 | 119 | 93 | 74 | 75 | 103 | 108 | 150 | 29 | 34 | 17 | 48 |
| Diff.ing SPG-2 | 0 | 0 | 0 | 10 | 104 | 104 | 96 | 32 | 136 | 62 | 126 | 54 | 56 | 39 | 65 |
| PreL | 0 | 0 | 0 | 11 | 132 | 113 | 90 | 28 | 97 | 98 | 107 | 45 | 76 | 34 | 72 |
| L | 0 | 0 | 0 | 33 | 232 | 159 | 157 | 37 | 225 | 197 | 128 | 127 | 55 | 114 | 171 |
| Z | 0 | 0 | 0 | 22 | 468 | 381 | 556 | 155 | 419 | 590 | 199 | 287 | 135 | 249 | 296 |
| PreP | 0 | 0 | 0 | 19 | 11 | 55 | 23 | 23 | 153 | 14 | 82 | 40 | 79 | 47 | 48 |
| P | 0 | 0 | 0 | 9 | 0 | 6 | 22 | 5 | 10 | 0 | 7 | 16 | 2 | 0 | 197 |
| D | 0 | 0 | 0 | 26 | 0 | 4 | 0 | 0 | 3 | 0 | 3 | 8 | 11 | 0 | 22 |
| SPC7 | 0 | 0 | 0 | 32 | 0 | 4 | 1 | 1 | 5 | 0 | 1 | 14 | 11 | 1 | 18 |
| S1 | 0 | 0 | 0 | 73 | 0 | 17 | 1 | 1 | 9 | 0 | 1 | 18 | 21 | 2 | 22 |
| S2 | 0 | 0 | 0 | 16 | 0 | 17 | 5 | 0 | 4 | 0 | 0 | 21 | 18 | 0 | 28 |
| S3 | 0 | 0 | 0 | 16 | 0 | 14 | 1 | 1 | 11 | 0 | 0 | 28 | 14 | 4 | 26 |
| Sertoli Cell | 203 | 76 | 26 | 2 | 53 | 5 | 3 | 12 | 7 | 1 | 3 | 15 | 163 | 104 | 9 |
| Leydig Cell | 123 | 79 | 103 | 30 | 12 | 80 | 25 | 165 | 27 | 40 | 87 | 60 | 68 | 99 | 29 |
| Endothelial Cell | 2 | 30 | 5 | 4 | 0 | 9 | 10 | 34 | 0 | 0 | 9 | 9 | 0 | 3 | 14 |
| PMC | 263 | 170 | 389 | 237 | 37 | 247 | 58 | 521 | 97 | 83 | 73 | 158 | 14 | 48 | 44 |
| T Cell | 8 | 5 | 9 | 40 | 6 | 22 | 12 | 51 | 2 | 2 | 29 | 1 | 3 | 5 | 1 |
| Macrophage | 27 | 10 | 23 | 29 | 21 | 29 | 14 | 67 | 7 | 21 | 25 | 17 | 7 | 14 | 3 |

C

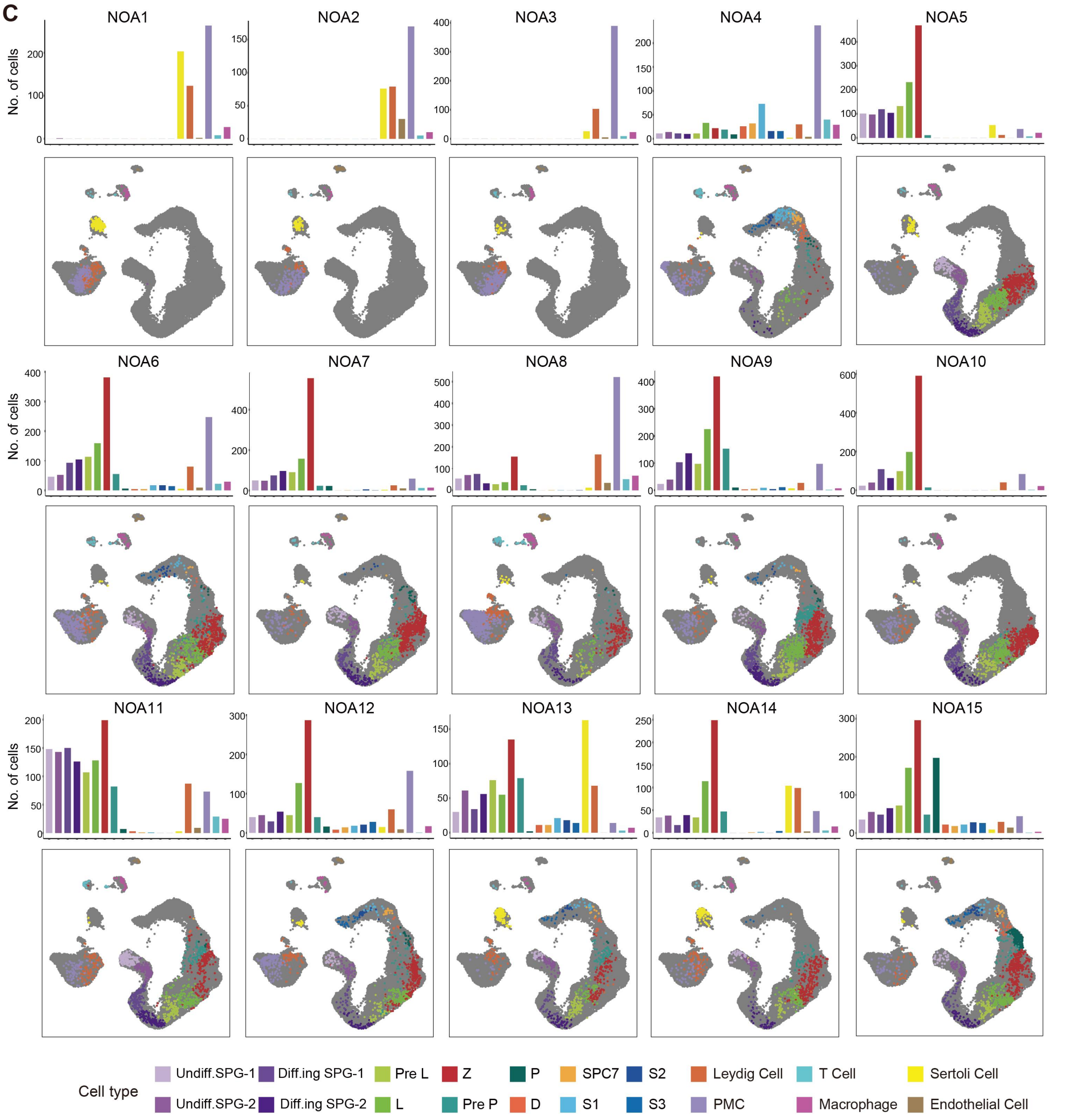


**Supplementary Fig.5 | Quality control of scATAC-seq data in NOA patients.**

**(A)**. Quality control metrics for each single cell in OA samples. Horizontal and vertical lines represent our quality standards: TSS enrichment >= 4; unique fragment count >= 10,000. **(B)**. Table of the number of germ cells in NOA patients. **(C)**.

UMAP visualization of all samples, colored by each cell type.

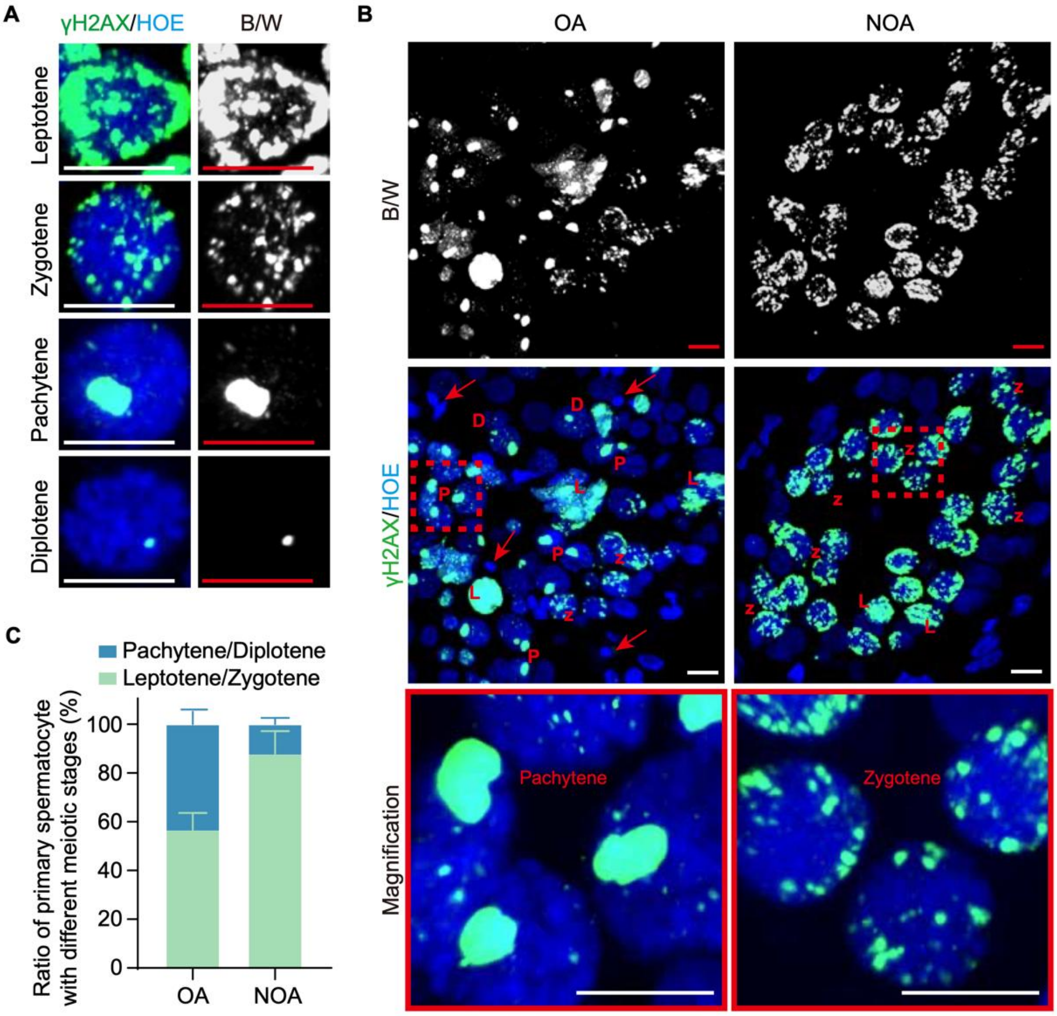


**Supplementary Fig.6 | γH2AX staining reveals meiotic arrest at the leptotene and zygotene stages in NOA patients.**

**(A)**. Representative images of γH2AX expression analysis during different meiotic stages in germ cells. γH2AX, green; HOE, blue. B/W, the green fluorescence of γH2AX was inverted to black/white (B/W) to highlight the meiotic stages in the right panel. **(B)**. γH2AX staining of human testicular tissues showed that primary spermatocytes in OA samples were at different developmental stages, including leptotene, zygotene, pachytene, and diplotene, and spermatids (arrow) were also observed. In contrast, most germ cells in NOA patients were arrested at the leptotene and zygotene stages. L, leptotene; Z, zygotene; P, pachytene; D, diplotene; S, spermatids. **(C)**. Counting the ratio of leptotene/zygotene and pachytene/diplotene germ cells in the testes of patients with OA and NOA (n = 5). All experiments were repeated for at least three times, and representative images are shown. Data are presented as the mean ± SD and analyzed by a two-tailed unpaired Student's t-test, n.s. P ⩾ 0.05 and ***P < 0.001. γ H2AX, green; Hoechst (HOE), blue. Scale bars, 10 μm (A and B).

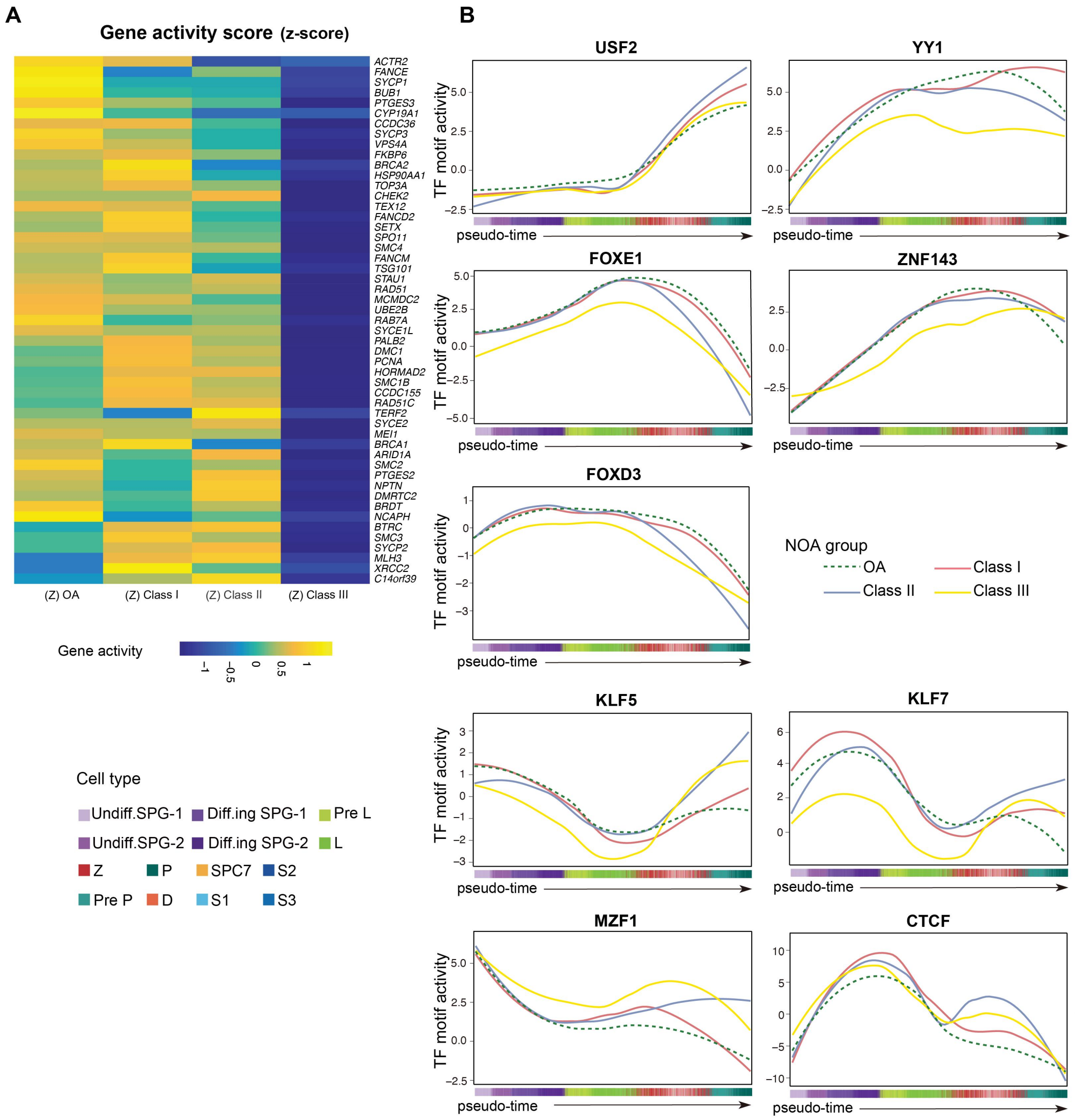


**Supplementary Fig.7 | Activation of key genes and critical TF motifs are disrupted in NOA patients.**

**(A)**. Heatmap depicting the differential gene activity between Class I-III patients and OA at Z stage. **(B)**. The trend of TFs motif activity score of some TFs in the OA and Class I-III patients along the pseudotemporal trajectory.

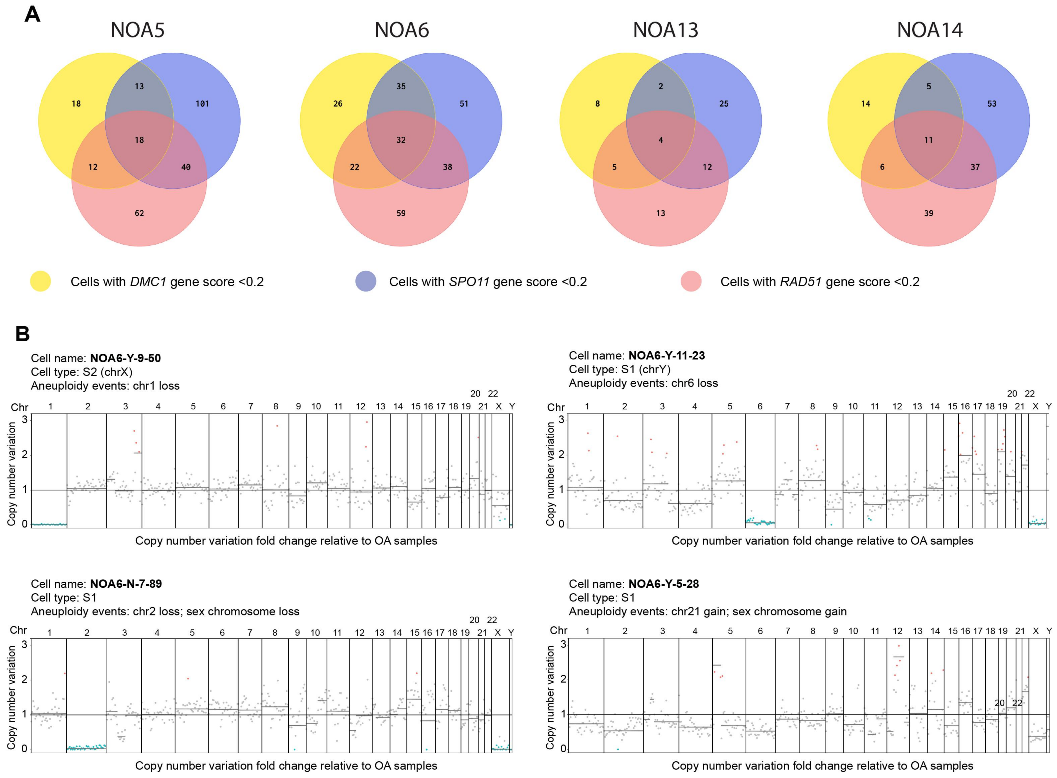


**Supplementary Fig.8 | Aneuploidy of sperm in NOA patients.**

**(A)**. Venn diagram showing the number of cells with activation scores below 0.2 for the *DMC1*, *SPO11*, and *RAD51* genes in NOA5, NOA6, NOA13, and NOA14. **(B)**. Visualization of the copy number variation (CNV) pattern in a single aneuploid cell from NOA6.

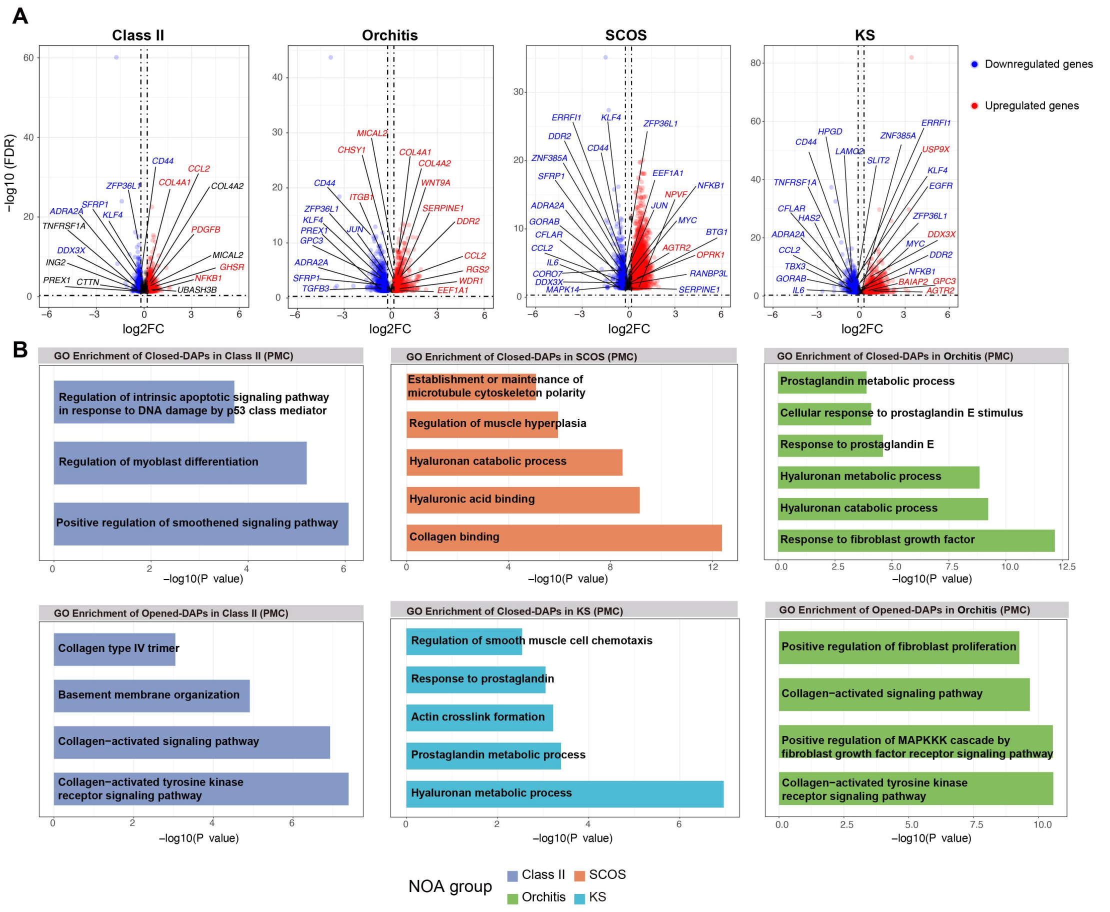


**Supplementary Fig.9 | PMC abnormalities in NOA patients.**

**(A)**. Volcano plot depicting the identification of upregulated activity (in red) and downregulated activity (in blue) genes in PMC across Class II, Orchitis, Sertoli cell-only syndrome (SCOS), and Klinefelter syndrome (KS). **(B)**. Functional enrichment for GO biological processes of the opened-DAPs and closed-DAPs in PMC across ClassII, Orchitis, SCOS and KS.

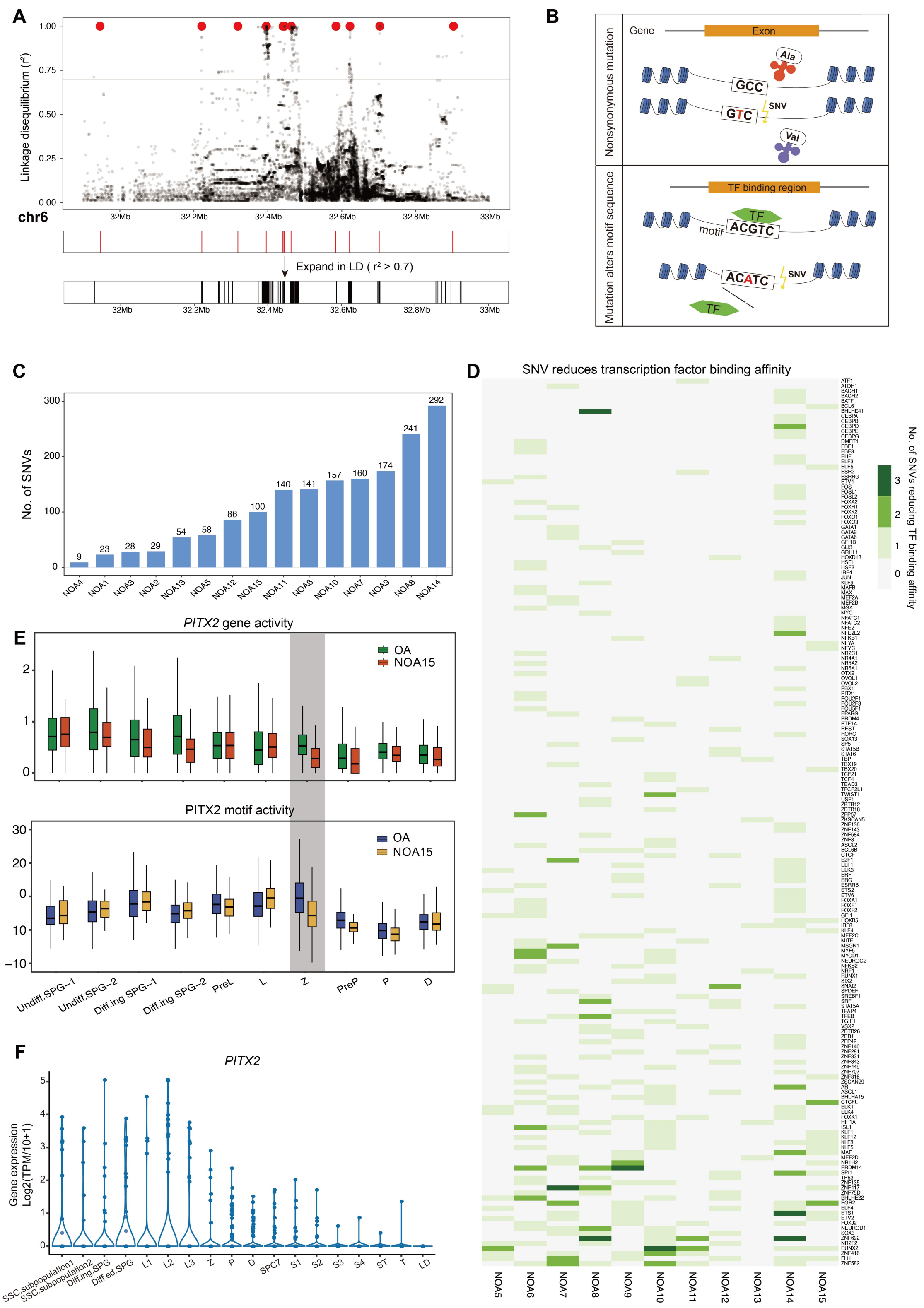


**Supplementary Fig.10 | GWAS loci and genomic variations in NOA patients.**

**(A)**. Compendium of GWAS loci within the MHC II region (n = 12) and LD-expanded polymorphisms. **(B)**. SNVs within active regulatory regions could directly impact TFs binding and alter protein sequences. **(C)**. The number of SNVs

identified in each patient. **(D)**. Heatmap illustrates SNVs changing TF binding motifs, reducing TF functional activity. **(E)**. Boxplot showing gene activity scores and TF motif activity scores of *PITX2* across spermatogenic stages from SPG to P. **(F)**. The transcriptional levels of *PITX2* at different stages of spermatogenesis.

**Table 1 |** Sample information.

**Table 2 |** Quality control information and metadata of all scATAC-seq.

**Table 3 |** Gene regulatory network of human spermatogenesis using Pando (abs(cor)>0.3).

**Table 4 |** Five classes of DSB-hotspot.

**Table 5 |** Coordinates of GWAS loci strongly associated with male infertility and their LD-expanded coordinates.
